# Distinct Patterns of Weight Gain, Age, and Subcortical Microstructure in Early Adolescence

**DOI:** 10.1001/jamanetworkopen.2025.22211

**Published:** 2025-07-23

**Authors:** Shana Adise, Zhaolong Adrian Li, Jonatan Ottino-González, Filip Morys, Peter A. Chiarelli, Tamara Hershey

**Affiliations:** 1 Division of Endocrinology, Diabetes and Metabolism, Department of Pediatrics, Children’s Hospital Los Angeles, Los Angeles, California; 2 Department of Psychiatry, Washington University in St Louis School of Medicine, St Louis, Missouri; 3 Montreal Neurological Institute, McGill University, Montreal, Quebec, Canada; 4 Department of Neurosurgery, Children’s Hospital Los Angeles, Los Angeles, California; 5 Department of Psychological and Brain Sciences, Washington University in St Louis, St Louis, Missouri; 6 Mallinckrodt Institute of Radiology, Washington University in St Louis, St Louis, Missouri; 7 Department of Neurology, Washington University in St Louis, St Louis, Missouri

## Abstract

**Importance:**

Associations between childhood obesity and brain microstructural differences have been observed. It remains unknown whether these associations are driven by sex-specific excessive weight gain. Restriction spectrum imaging characterizes brain tissue microstructural health via water diffusion, where the restricted normalized isotropic (RNI) compartment assesses neuronal and glial cellularity, which may reflect neuroinflammation, synaptic pruning, or both.

**Objective:**

To identify associations among RNI scaling factor values, normal neurodevelopment, and weight gain during early adolescence.

**Design, Setting, and Participants:**

This cohort study used data from the Adolescent Brain Cognitive Development (ABCD) Study, a 10-year, ongoing, multisite longitudinal cohort study conducted among youths aged 9 to 20 years. The analyses focused on data from baseline (collected in 2016-2018) and the 2-year follow-up (collected in 2018-2020). Participants who initially had healthy weight (body mass index [BMI] percentile <85th) at age 9 to 10 years were eligible for this study. Data analysis was performed between March 2024 and March 2025.

**Main Outcomes and Measures:**

Linear mixed-effects models were used to examine bidirectional associations among RNI, age, and BMI (a proxy for weight gain) across 16 appetite-controlling brain regions. First, analysis was performed among youths with healthy-weight, weight-stable (HW-WS) status, stratified by sex. Second, an evaluation was conducted to determine how these associations changed among all youths (eg, conversion to healthy-weight, non–weight-stable [HW-NS] status).

**Results:**

At baseline, data were available for 3110 youths (mean [SD] baseline age, 119.2 [7.5] months); at year 2, only 1855 youths had complete data. Of these 1855 youths, 1072 (592 males [55.2%]) had HW-WS and 773 (445 females [57.2%]) had HW-NS. Among youths with HW-WS, RNI values were associated with age but not BMI. Among youths with varying weight gain, RNI values had bidirectional associations with BMI across many subcortical regions independent of age. In females, but not in males, higher RNI values had robust associations with greater increases in BMI over time.

**Conclusions and Relevance:**

In this cohort study, RNI values were associated with age and BMI, and greater RNI values beyond normal developmental processes may suggest neuroinflammation. Thus, higher RNI values may signal neuroinflammatory processes associated with unhealthy weight gain, suggesting potential for the RNI scaling factor as an early indicator of obesity-related neurodevelopmental changes in adolescence. Future mechanistic studies are needed to determine the specific cellular changes underlying these associations.

## Key Points

**Question:**

Are restricted normalized isotropic (RNI) scaling factor values, a potential marker of neuroinflammation and synaptic pruning, in brain subcortical appetite-related regions associated with body mass index (BMI)?

**Findings:**

In this cohort study of 3110 youths, RNI values were associated with being older but not with BMI; however, RNI values had positive bidirectional associations with weight gain at the 2-year follow-up. In females, higher RNI values were associated with greater weight gain over a 2-year period.

**Meaning:**

These findings suggest that higher RNI values may reflect neuroinflammatory processes associated with excessive weight gain, suggesting its potential as an early indicator of obesity-related neurodevelopmental changes.

## Teaser

This cohort study uses the restricted normalized isotropic scaling factor to investigate bidirectional associations among neurodevelopment, subcortical microstructure, and weight gain among youths.

## Introduction

Obesity and its comorbidities are leading preventable causes of mortality.^1^ Known clinical sequelae of obesity include heart disease, increased stroke risk, and cancer,^2^ although the extent to which it affects brain development is less understood. Studies in animals and older adults are beginning to suggest a strong correlation between obesity and cognitive decline,^3,4,5,6,7^ including early-onset dementia.^8,9^ Compared with their counterparts with healthy weight, children with obesity have less optimal cognitive^10,11,12,13^ and brain outcomes,^14,15,16,17,18,19,20,21^ and these differences may occur prior to obesity onset.^22,23^ The neurobiological mechanisms driving these associations are unclear, especially during adolescence, a period when excessive weight gain risk is high^24^ and the brain is undergoing rapid development.^25^ Because weight gain may have avoidable long-term cognitive consequences, it is imperative to understand how weight gain may affect neurodevelopment.

Prior human adult studies and animal models have suggested that the association between weight and cognitive decline may be partially explained by diet- or weight-induced neuroinflammation.^26^ Although acute neuroinflammation is not harmful, chronic overactivation of this system can erode myelin,^27^ induce cell death, and alter brain structure.^26^ Consequently, this may be one reason why obesity is associated with differences in brain structure in adolescence.^15,28^ Neuroinflammation can occur prior to weight gain,^29^ and it may first affect regions that are directly involved with appetite control (eg, the hypothalamus or hippocampus).^30^ Thus, any changes to these regions may dysregulate appetite control and cause some individuals to overeat and gain weight. To date, no study has investigated whether neuroinflammation is associated with bidirectional associations and weight gain in adolescents.

Modern diffusion-based magnetic resonance imaging (MRI) methods have allowed insight into potential neuroinflammation through assessment of tissue microstructure, a marker of brain health. One such approach, restriction spectrum imaging (RSI), models water diffusion within different anatomic compartments to assess tissue microstructure. RSI estimates the restricted normalized isotropic (RNI) scaling factor, which measures spherical water diffusion within neuronal and glial cell bodies, providing an in vivo value that is positively correlated with tissue cellularity (eg, glial cells, neuronal cell bodies)^31^; larger RNI values have been postulated to indicate a neuroinflammatory phenotype in some contexts,^15,32^ although histologic confirmation remains necessary. Larger RNI values during adolescence may also reflect normative developmental processes,^33^ such as synaptic pruning and increased myelination, where glial cells play an active role.^34,35^ Pruning is a developmental process through which unnecessary neurons and synapses are removed to favor more efficient neural circuits, and glial cells play an active role in this. Yet previous interpretations of the RNI scaling factor and neurodevelopment^33^ were based on a sample in which approximately 33% of participants had overweight or obesity, making it difficult to distinguish age- vs weight-induced microstructure associations.

In this study, we examined associations among neurodevelopment, microstructure, and weight gain using RNI values among a sample of youths enrolled in the Adolescent Brain Cognitive Development (ABCD) Study.^36^ We aimed to capture the natural progression of these associations by focusing on youths who initially had healthy weight (as defined by the CDC)^37^ at study onset. We also examined whether weight gain was associated with microstructural changes beyond typical neurodevelopmental associations of age and puberty. Because neuronal maturation trajectories are confounded by sex-specific pubertal effects,^38,39^ we examined these associations separately by sex. Importantly, we focused on subcortical regions of the brain because they are directly involved in food intake regulation^40,41^ and variations among their structure and function have been associated with overeating and weight gain.^15,16,19,20,21,42,43^ We hypothesized that greater RNI values would be associated with greater weight gain independent of age over time but that these associations would be more robust in females than males due to differences in pubertal onset and in line with previous findings in this age range.^23^

## Methods

### Study Design and Participants

This cohort study used data from the ABCD Study, a 10-year, 21-site consortium study that enrolled youths (ages 9-10) between 2016 and 2018. This diverse sample (n = 11 878) was recruited to match the American Community Survey demographics. Study recruitment, objectives, design, and assessments have been published in multiple articles^44,45,46,47^ and on the ABCD Study website.^36^ Each study site obtained caregiver consent and youth assent, and a centralized institutional review board at the University of California, San Diego approved all protocols. For this study, we used the 4.0 data release^48^ and assessments at baseline (2016-2018) and year 2 (2018-2020). Youths with a healthy weight at baseline (body mass index [BMI] percentile <85th^37^) were eligible for the study. Complete methods and exclusion criteria are presented in the eMethods and eTable 1 in Supplement 1, respectively. This study followed the Strengthening the Reporting of Observational Studies in Epidemiology (STROBE) reporting guideline.

### Demographics

Caregivers reported on each youth’s race and ethnicity, sex at birth, date of birth, family structure, and their own highest household level of education. Race and ethnicity data were collected for descriptive purposes to determine generalizability. There were 16 categories for race (in eMethods in Supplement 1) that were collapsed into a 6-item variable for descriptive purposes only; as such, race is reported as American Indian or Alaska Native or Native Hawaiian or Other Pacific Islander, Asian, Black, White, other race (not specified), or multiple races; ethnicity is reported as Hispanic or non-Hispanic.

### Physical Assessments

Height and weight were converted to BMI (calculated as body weight in kilograms divided by height in meters squared) and the Centers for Disease Control and Prevention^37^ BMI *z* scores and percentiles (for descriptive purposes only^49^); raw BMI was used in analyses. Puberty was assessed by caregiver and youth reports.^50^ Additional details are reported in the eMethods in Supplement 1.

### Image Acquisition and Preprocessing

The ABCD Study Data Analytics Core preprocessed MRI scans.^33,51^ RSI was fitted to diffusion-weighted imaging data to extract RNI diffusion estimates, which reflect water diffusion in cell bodies (eg, glial cells, neurons)^28^ and are elevated in obesity and neuroinflammatory states.^15,21,27,28,52,53,54,55^ RNI diffusion estimates for 8 bilateral subcortical regions of interest were extracted using the Aseg Atlas (FreeSurfer) to garner estimates in the bilateral accumbens, amygdala, caudate, hippocampus, thalamus, pallidum, putamen, and ventral diencephalon, all associated with appetite regulation.^40,41^

### Classification of Weight and Weight Stability

We classified youths as having healthy-weight, weight-stable (HW-WS) status if they had healthy weight (ie, BMI percentile <85th^37^) and an SD of less than 1 for within-individual BMI variation from baseline through year 2. Youths with missing year 2 data were not eligible for group classification. A separate group of participants were partitioned based on (1) healthy weight at study baseline and (2) conversion to non–weight-stable status (HW-NS) during the study period. HW-NS status was defined as an individual-specific increase in BMI of 1 SD or more, demonstrated overweight or obesity (ie, BMI percentile ≥85th^3^), or both by year 2. Group classification was used to explore whether this grouping could be a meaningful risk factor for RNI-related differences.

### Statistical Analysis

#### Linear Mixed-Effects Models

Analyses using linear mixed-effects models were conducted in Python (Python Software Foundation) using pymer4, version 0.8.2^56,57^ (the model framework is discussed next), and were corrected using the Benjamini-Hochberg approach (n = 16 tests). Covariates were selected based on their known associations with BMI, highest caregiver education (effects coded), MRI motion (eg, framewise displacement), and neurodevelopment (eg, age). Age in months was modeled to account for developmental differences and the variation in time between study visits. Crossed random effects for MRI serial number (eg, MRI scanner identifier [ID]) and participant ID were also included. Analyses were stratified by sex, given known differences in maturational trajectories.^38,39^

#### Model Framework

To test our research questions, we used a tiered approach across several models. First, we replicated the analyses by Palmer et al^33^ but focused only on youths with HW-WS to establish the association between age (eg, main effect) and RNI values without confounding associations of weight gain (model 1). Changes over time between BMI (main effect) and an interaction term (BMI × Age interaction) were modeled while also accounting for the confounding associations of puberty. Because previous literature suggests that smaller subcortical volumes may precede greater BMI increases from age 9 to 12 years,^23^ we also examined whether RNI values were prospectively associated with changes in BMI over time (model 2) and whether this association was moderated by age (ie, interaction effects [RNI × Age interaction]), independent of puberty. Together, models 1 and 2 assessed reciprocal associations: that is, whether BMI was associated with changes in RNI values over time (model 1) and whether RNI values were associated with changes in BMI over time (model 2). Although these models did not establish causality, they provided insights into how these variables may relate to each other across development. Note, analyses were stratified by sex to allow for clear interpretation of potential sex differences. The model specification is as follows:

Model 1: RNI Scaling Factor = β0 + β1(Age) + β2(BMI) + β3(Age × BMI) + β4(Puberty) + β5(Caregiver Education) + β6(Framewise Displacement) + (1∣Subject ID) + (1∣MRI Scanner ID) + ϵ

Model 2: BMI = β0 + β1(Age) + β2(RNI) + β3(Age × RNI) + β4(Puberty) + β5(Caregiver Education) + β5(Framewise Displacement) + (1∣Subject ID) + (1∣MRI Scanner ID) + ϵ

Next, we explored how our outcomes of interest differed across a weight gain continuum. Models 1 and 2 were rerun for all youths who had healthy weight at baseline to account for how varying degrees of weight gain related to RNI. Finally, a group classification (HW-WS vs HW-NS) was used to explore whether this grouping could be a meaningful risk factor for RNI-related differences. Further details are provided in the eResults and eDiscussion in Supplement 1.

Two-tailed *P* < .05 was considered significant. Statistical analysis was performed between March 2024 and March 2025 with Python using pymer4, version 0.8.2.

## Results

### Descriptive Statistics

**Figure 1.**
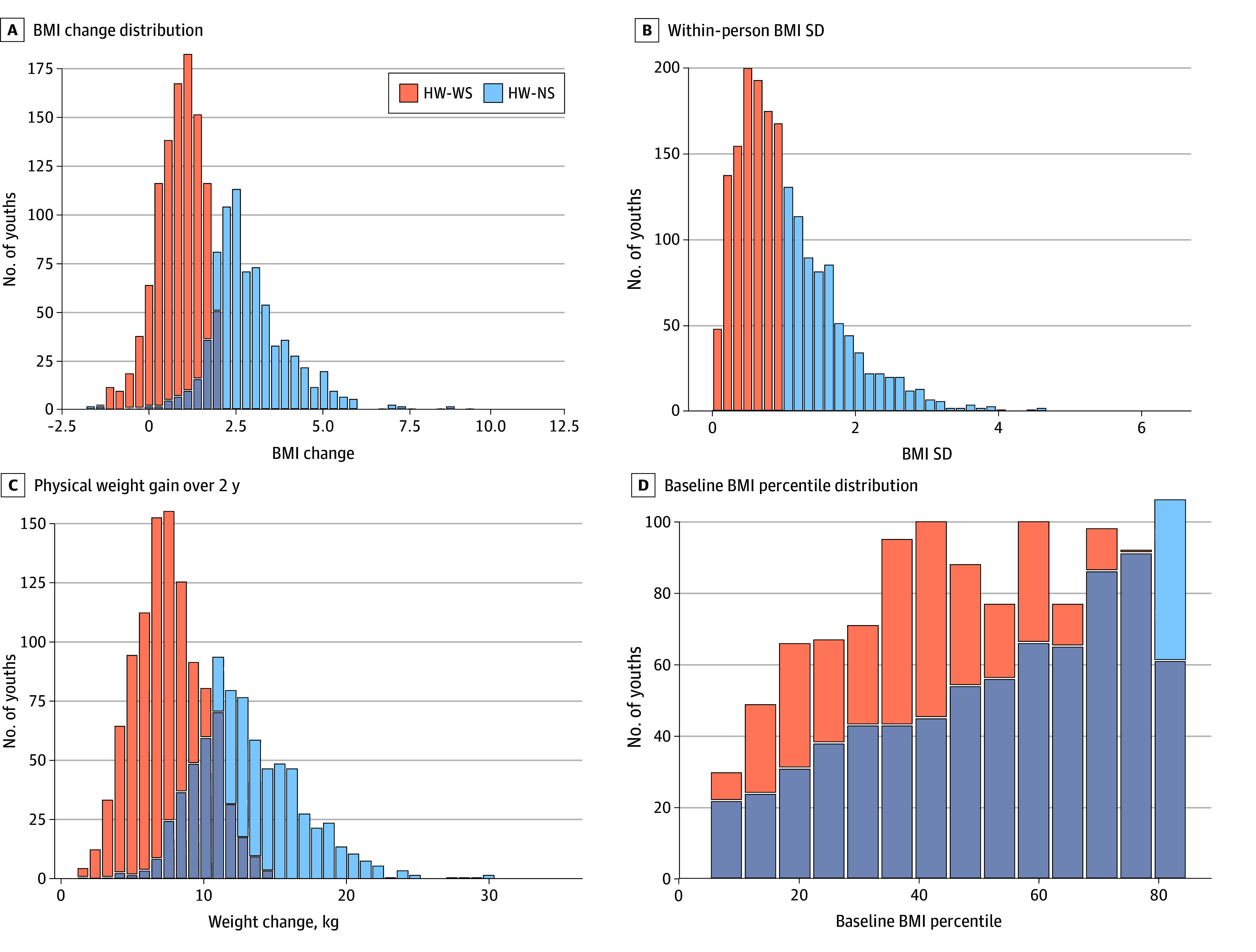
Body Mass Index (BMI) and Weight Distributions for Youths in the Healthy-Weight, Weight-Stable (HW-WS) and Healthy-Weight, Nonstable (HW-NS) Groups A, Distribution of BMI change (BMI at year 2 − BMI at baseline) by group membership. B, Within-person BMI SD, which was used as a proxy for weight gain, as the SD represents how much each individual’s BMI varied over the 2-year period. C, How much physical weight was gained over the 2-year period in kilograms. D, Distribution of baseline BMI percentiles by weight group, which highlights that HW-WS and weight group membership were not dependent on the starting point of BMI percentile.

A total of 3110 youths had available data at baseline, but only 1855 had complete data at year 2 (Table 1). Of these 1855 youths, 1072 met the HW-WS criterion (480 females [44.8%] and 592 [55.2%] males; mean [SD] age at baseline vs year 2, 118.1 [7.1] vs 141.9 [7.1] months) and 773 (445 females [57.2%] and 328 males [42.4%]; mean [SD] age at baseline vs year 2, 119.2 [7.2] vs 143.1 [7.3] months) met the HW-NS criterion. With regard to race and ethnicity, 5 youths (0.5%) in the HW-WS group identified as American Indian or Alaska Native or as Native Hawaiian or Other Pacific Islander, 23 (2.2%) as Asian, 77 (7.3%) as Black, 142 (13.2%) as Hispanic, 821 (76.6%) as White, 30 (2.8%) as other race, and 107 (10.1%) as multiple races; these data were missing for 9 youths (0.8%). In the HW-NS group, 3 youths (0.4%) identified as American Indian or Alaska Native or as Native Hawaiian or Other Pacific Islander, 17 (2.2%) as Asian, 85 (11.0%) as Black, 147 (19.0%) as Hispanic, 564 (73.0%) as White, 34 (4.4%) as other race, and 63 (8.2%) as multiple races; these data were missing for 7 youths (0.9%). Visualization of group membership is illustrated in Figure 1 and eFigure 1 in Supplement 1. Demographic data are presented in Table 2 and eTables 2 to 4 in Supplement 1.

**Table 1.** Demographic Data for All Youths With Healthy Weight at Baseline, Stratified by Sex^a^

Characteristic	All youths (N = 3110)	Females (n = 1546)	Males (n = 1564)	*P* value^b^
Sex				
Female	1546 (49.7)	1546 (100)	NA	NA
Male	1564 (50.3)	NA	1564 (100)	
Education				
Less than high school	75 (2.4)	42 (2.7)	33 (2.1)	.70
High school or GED	167 (5.4)	79 (5.1)	88 (5.6)	
Some college	641 (20.6)	310 (20.1)	331 (21.2)	
Bachelor’s degree	892 (28.7)	449 (29.0)	443 (28.3)	
Postgraduate education	1335 (42.9)	666 (43.1)	669 (42.8)	
Income, $				
<50 000	583 (18.7)	299 (19.3)	284 (18.2)	.28
50 000-100 000	844 (27.1)	424 (27.4)	420 (26.9)	
>100 000	1489 (47.9)	739 (47.8)	750 (48.0)	
Missing	194 (6.2)	84 (5.4)	110 (7.0)	
Race^c^				
American Indian or Alaska Native or Native Hawaiian or Other Pacific Islander	16 (0.5)	9 (0.6)	7 (0.4)	.92
Asian	67 (2.2)	37 (2.4)	30 (1.9)	
Black	298 (9.6)	141 (9.1)	157 (10.0)	
White	2289 (73.6)	1144 (74.0)	1145 (73.2)	
Other	100 (3.2)	50 (3.2)	50 (3.2)	
Multiple races	316 (10.2)	153 (9.9)	163 (10.4)	
Missing	24 (0.8)	12 (0.8)	12 (0.8)	
Ethnicity^d^				
Hispanic	465 (15.0)	250 (16.2)	215 (13.7)	.17
Non-Hispanic	2612 (84.0)	1280 (82.8)	1332 (85.2)	
Missing	33 (1.1)	16 (1.0)	17 (1.1)	
Weight class, healthy weight^e^	3110 (100)	1546 (100)	1564 (100)	>.99
Age, mean (SD), mo	119.2 (7.5)	119.2 (7.5)	119.2 (7.5)	.99
Puberty, mean (SD)	1.9 (0.8)	2.2 (0.9)	1.6 (0.5)	<.001
BMI, mean (SD)	16.9 (1.4)	16.9 (1.5)	16.8 (1.3)	.02
Weight, mean (SD), kg	33.0 (4.9)	33.3 (5.3)	32.8 (4.5)	.02
Weight gain, mean (SD), kg^f^	9.9 (4.2)	10.7 (4.2)	9.1 (4.0)	<.001
Income-to-needs ratio, mean (SD)^g^	4.2 (2.3)	4.2 (2.3)	4.2 (2.4)	.95
Household poverty rate, mean (SD)^h^	27 716.1 (5400.6)	27 668.3 (5442.1)	27 763.4 (5360.6)	.63

Abbreviations: BMI, body mass index (calculated as weight in kilograms divided by height in meters squared); GED, generalized education degree; NA, not applicable.

^a^ Unless otherwise indicated, values are presented as the No. (%) of youths.

^b^ *P* values reflect χ^2^ and *t* tests where appropriate.

^c^ Race (16 categories, collapsed into 6 groups) was self-reported by the caregiver. Descriptive statistics are reported for self-reported race only for interpretation of sample diversity.

^d^ Ethnicity (2 categories) was self-reported by the caregiver.

^e^ Weight class was determined using the 2000 Centers for Disease Control and Prevention growth charts.

^f^ Missing for 1259 youths.

^g^ Missing for 216 youths.

^h^ Missing for 38 youths.

**Table 2.** Demographic Data for Youths With HW-WS or HW-NS Status at 2-Year Follow-Up^a^

Characteristic	HW-WS group (n = 1072)	HW-NS group (n = 773)	*P* value^b^
Sex			
Female	480 (44.8)	445 (57.2)	<.001
Male	592 (55.2)	328 (42.4)	
Education			
Less than high school	21 (2.0)	21 (2.7)	<.001
High school or GED	42 (3.9)	57 (7.4)	
Some college	202 (18.8)	186 (24.1)	
Bachelor’s degree	326 (30.4)	202 (26.1)	
Postgraduate education	481 (44.9)	307 (39.7)	
Income, $			
<50 000	289 (27.0)	235 (30.4)	<.001
50 000-100 000	173 (16.2)	169 (21.9)	
>100 000	548 (51.2)	318 (41.1)	
Missing	62 (5.8)	51 (6.6)	
Race^c^			
American Indian or Alaska Native or Native Hawaiian or Other Pacific Islander	5 (0.5)	3 (0.4)	.02
Asian	23 (2.2)	17 (2.2)	
Black	77 (7.3)	85 (11.0)	
White	821 (76.6)	564 (73.0)	
Other	30 (2.8)	34 (4.4)	
Multiple races	107 (10.1)	63 (8.2)	
Missing	9 (0.8)	7 (0.9)	
Ethnicity^d^			
Hispanic	142 (13.2)	147 (19.0)	.001
Non-Hispanic	930 (86.8)	626 (81.0)	
Weight class^e^			
Baseline			
Healthy weight^f^	1072 (100)	773 (100)	>.99
Year 2 follow-up			
Healthy weight^g^	1072 (100)	556 (71.9)	<.001
Overweight	NA	201 (26.0)	
Obesity	NA	16 (2.1)	
Age, mean (SD), mo			
Baseline	118.1 (7.1)	119.2 (7.2)	.001
Year 2	141.9 (7.1)	143.1 (7.3)	<.001
Puberty, mean (SD)			
Baseline	1.8 (0.7)	2.0 (0.8)	<.001
Year 2	2.4 (0.9)	2.8 (1.0)	<.001
BMI, mean (SD)			
Baseline	16.7 (1.3)	17.2 (1.4)	<.001
Year 2	17.5 (1.4)	20.2 (2.1)	<.001
Weight, mean (SD), kg			
Baseline	32.3 (4.4)	34.1 (5.0)	<.001
Year 2	39.9 (5.7)	47.2 (7.5)	<.001
Weight gain, mean (SD), kg	7.6 (2.5)	13.2 (3.9)	<.001
Income-to-needs ratio, mean (SD)^h^	4.4 (2.4)	3.9 (2.3)	<.001
Household poverty rate, mean (SD)^i^	27 629.1 (5187.1)	27 722.3 (5808.6)	.63

Abbreviations: BMI, body mass index (calculated as weight in kilograms divided by height in meters squared); GED, generalized education degree; HW-NS, healthy weight, non–weight stable; HW-WS, healthy weight, weight stable; NA, not applicable.

^a^ Unless otherwise indicated, values are presented as the No. (%) of youths. Although 1855 youths had available data at year 2, 10 youths (7 males and 3 females) were excluded from the HW-WS group because their weight status classification fluctuated between healthy weight to overweight to healthy weight from baseline to year 2. The HW-NS grouping variable was used for the logistic regression analyses only.

^b^ *P* values reflect χ^2^ and *t* tests where appropriate.

^c^ Race (16 categories, collapsed into 6 groups) was self-reported by the caregiver. Descriptive statistics are reported for self-reported race only for interpretation of sample diversity.

^d^ Ethnicity (2 categories) was self-reported by the caregiver.

^e^ Weight class was determined using the 2000 Centers for Disease Control and Prevention growth charts.

^f^ Baseline (ages 9-10 years; healthy weight defined as BMI percentile <85%).

^g^ Year 2 follow-up (ages 11-12 years; obesity or overweight defined as BMI percentile ≥85%).

^h^ Missing for 125 youths.

^i^ Missing for 21 youths.

By year 2, 217 of the 1855 youths (11.7%) transitioned from initial healthy weight to having overweight or obesity. Ten youths (7 males and 3 females) were excluded from the HW-WS group because their weight fluctuated between overweight and healthy weight between years 1 and 2. Youths in the HW-WS group gained a mean (SD) of 7.6 (2.5) kg (over 2 years) compared with 13.2 (3.9) kg in the HW-NS group. Notably, by year 2, 217 of the 773 youths (28.1%) in the HW-NS group transitioned to having overweight or obesity (Table 2); 556 youths (71.9%) remained in the healthy weight category even though they did not have stable weight. This finding suggests that using weight classification cutoffs (eg, healthy weight, overweight, or obesity) solely as markers for metabolic consequences may overlook unhealthy weight gain among youths in the healthy weight category.^49^ As such, it may be more insightful to examine within-person changes in BMI, rather than changes in weight categories, to determine weight stability.

### Associations Stratified by Sex

#### Males With HW-WS

**Figure 2.**
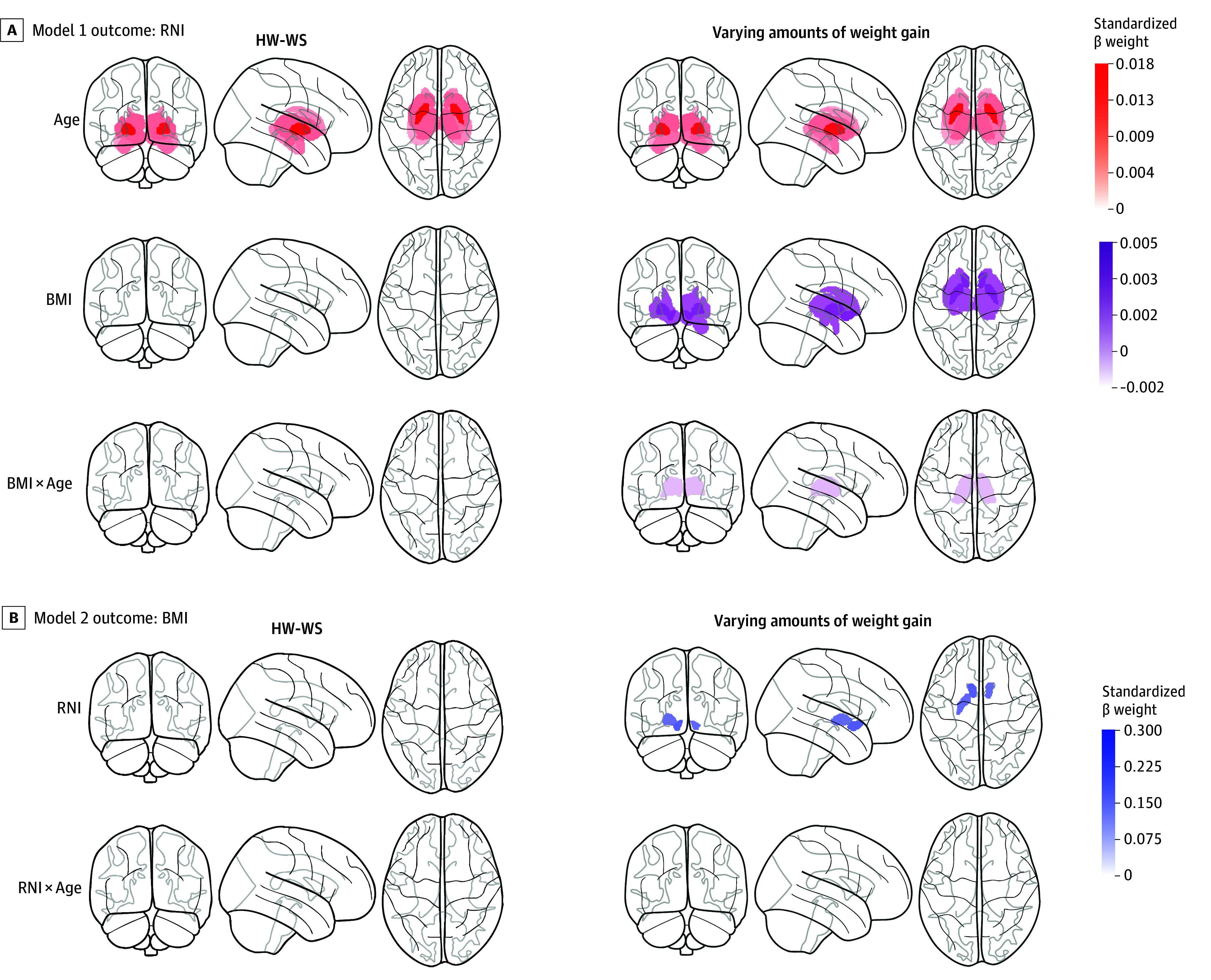
Associations Between BMI and Restricted Normalized Isotropic (RNI) Values for Male Youths Results are shown for analyses that included youths who met the healthy-weight, weight-stable (HW-WS) criterion (left column) and across the BMI continuum (right column) (ie, had a healthy weight at baseline but may have gained relatively unhealthy weight). A, Model 1 outcome. The left and right panels correspond to model 1 (BMI × Age → RNI) examining whether BMI over time (ie, baseline to year 2) was associated with changes in RNI values (outcome variable, ie, dependent variable). B, Model 2 outcome. The left and right panels correspond to model 2 (RNI × Age → BMI) examining whether changes in RNI values over time (ie, baseline to year 2) were associated with changes in BMI (outcome variable, ie, dependent variable). Brain figures indicate associations (corrected for multiple comparisons) with each main effect and interaction. Models controlled for highest household education level (effects coded), motion during the diffusion-weighted imaging scan and random effects modeled for scanner ID and subject ID. β weights for each of the regions can be found in eTables 5 to 8 in Supplement 1. The color bars show the standardized β weight for each region of interest (ROI). BMI indicates body mass index (calculated as weight in kilograms divided by height in meters squared).

Results of model 1 (outcome: RNI) revealed that age was positively associated with RNI values across the entire subcortex. There were no main effects of BMI or 2-way interactions of BMI × Age on RNI values (Figure 2A), and puberty was not associated with RNI values (β weights in eTable 5 in Supplement 1). Results of model 2 (outcome: BMI) suggested that age and puberty were associated with greater BMI (ie, older males were heavier: β = 0.40 [95% CI, 0.34-0.42], *P* < .001; higher pubertal scores were associated with being heavier: β = 0.10 [95% CI, 0.03-0.17], *P* < .05), but there were no associations between RNI values and BMI or between RNI × Age on BMI (Figure 2B; β weights in eTable 6 in Supplement 1).

#### Males With Variable Weight Gain

After establishing reference associations between RNI values and BMI in males with HW-WS, we interrogated how these associations changed with varying amounts of weight gain, including those who had transitioned to having overweight or obesity or remained in the healthy weight category but were not weight stable. Results from model 1 (outcome: RNI values) suggested that after controlling for associations with age and puberty on RNI values, BMI was positively associated with RNI values in the bilateral accumbens, caudate, pallidum, putamen, thalamus, ventral diencephalon, and right amygdala, and a 2-way interaction (BMI × Age) in the bilateral thalamus (Figure 2A; β weights in eTable 7 in Supplement 1) was observed.

Results from model 2 (outcome: BMI) suggested that after controlling for the association of age and puberty on BMI, RNI values in the bilateral accumbens and left pallidum were positively associated with greater BMI (Figure 2B). The association between age and BMI is illustrated in eFigure 4 in Supplement 1. No RNI × Age interactions were observed (β weights in eTable 8 in Supplement 1).

#### Females With HW-WS

**Figure 3.**
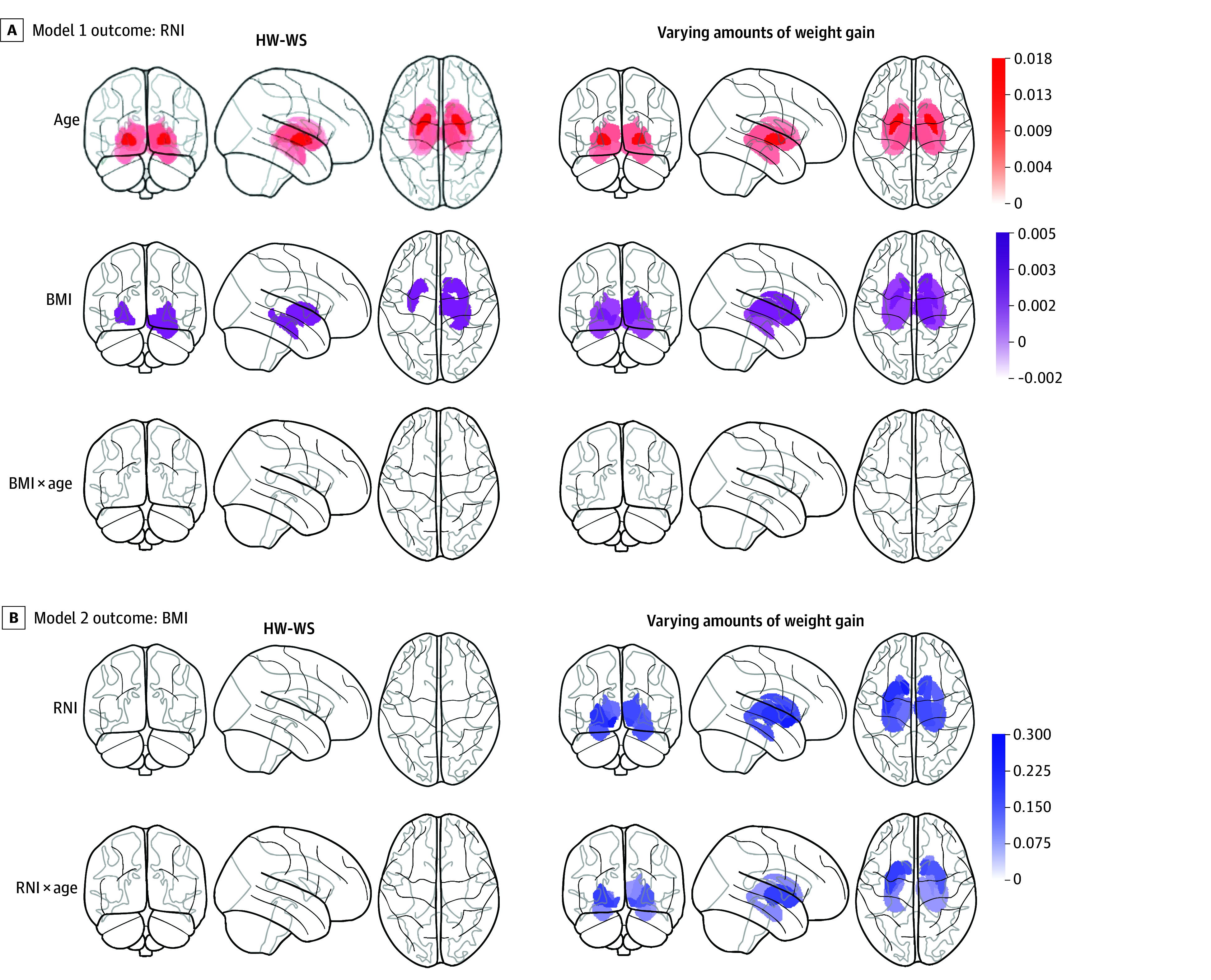
Associations Between BMI and Restricted Normalized Isotropic (RNI) Values for Female Youths Results are shown for analyses that included youths who met the healthy-weight, weight-stable (HW-WS) criterion (left column) and across the BMI continuum (right column) (ie, had a healthy weight at baseline but may have gained relatively unhealthy weight). A, Model 1 outcome. The left and right panels correspond to model 1 (BMI × Age → RNI) examining whether BMI over time (ie, baseline to year 2) was associated with changes in RNI values (outcome variable; ie, dependent variable). B, Model 2 outcome. The left and right panels correspond to model 2 (RNI × Age → BMI) examining whether changes in RNI values over time (ie, baseline to year 2) were associated with changes in BMI (outcome variable; ie, dependent variable). Brain figures indicate associations (corrected for multiple comparisons) with each main effect and interaction. Models controlled for highest household education level (effects coded), motion during the diffusion-weighted imaging scan and random effects modeled for scanner identifier (ID) and subject ID. β weights for each of the regions can be found in eTables 13 to 16 in Supplement 1. The color bars show the standardized β weight for each region of interest (ROI). BMI indicates body mass index (calculated as weight in kilograms divided by height in meters squared).

Results of model 1 (outcome: RNI) suggested that age was positively associated with RNI values across the entire subcortex. After controlling for the association of age and puberty on RNI, among females with HW-WS, BMI was positively associated with RNI values across the right hippocampus as well as the ventral diencephalon and bilateral putamen. No BMI × Age interactions were observed (Figure 3A; β weights in eTable 9 in Supplement 1). Results of model 2 (outcome: BMI) suggested that age and puberty were associated with greater BMI (ie, older females were heavier: β = 0.50 [95% CI, 0.31-0.42], *P* < .001; females with more advanced puberty were heavier: β = 0.17 [95% CI, 0.12-0.24], *P* < .001) but there were no associations between RNI values and BMI nor between RNI × Age on BMI among females with HW-WS (Figure 3B; β weights in eTable 10 in Supplement 1).The association between age and BMI is illustrated in eFigure 4 in Supplement 1.

Sensitivity analyses were run due to a discrepancy between BMI and RNI values for models 1 and 2, possibly attributable to the confounding associations of age on RNI values and BMI. To account for the fact that heavier females were older, we residualized age on BMI and reran the model to determine whether the associations of model 1 were valid. Residualizing age on BMI indicated that the associations between BMI and RNI values (model 1) no longer remained. Therefore, we interpreted these associations to suggest that among females with HW-WS, being heavier was not associated with greater RNI values and vice versa.

#### Females With Variable Weight Gain

Models were rerun to interrogate how these associations changed when including females with various amounts of weight gain, including those who had transitioned to have overweight or obesity or did not have stable weight but remained in the healthy weight category. Results from model 1 (outcome: RNI) suggested that independent of age and puberty, BMI was positively associated with RNI values across all regions of interest except the amygdala (Figure 3A; β weights in eTable 11 in Supplement 1). Results of model 2 (outcome: BMI) suggested a similar pattern, yet there were 2-way associations between RNI × Age on BMI in the bilateral accumbens, amygdala, caudate, hippocampus, pallidum, putamen, and right thalamus (Figure 3B; β weights in eTable 12 in Supplement 1). This finding suggests that greater RNI values over time may be associated with greater weight gain in females.

### Group Classification and Interactions With Sex

Group classification (HW-WS vs HW-NS) findings are reported in the eResults, eFigures 2 and 3, and eTables 13 to 20 in Supplement 1. Sex differences and interactions with sex (ie, 3-way interactions) are reported in eTables 21 to 24 in Supplement 1.

## Discussion

This study examined associations among BMI, age, puberty, and subcortical microstructure via the RNI scaling factor (an indirect estimate of aggregate neuronal/glial cellularity) by sex in a large cohort study of youths with healthy weight at study onset. We first analyzed these associations among youths with HW-WS, establishing reference trajectories in the absence of unstable weight gain. In the HW-WS group, being older was associated with greater RNI values but not BMI. As youths with nonstable weight gain (HW-NS) were added into the analyses, beyond any associations of age (and puberty), BMI was positively, bidirectionally, and dissociably associated with RNI values. Additionally, over time, greater RNI values corresponded with greater weight gain in females. These results suggest that greater RNI values may reflect normal developmental processes^33^ and/or the consequences of (unhealthy) weight gain (eg, neuroinflammation).

Using HW-WS status, we established references for associations between weight and RNI values and age and RNI values. Our findings, like those of Palmer et al,^33^ showed robust associations between age and RNI values but without the confound of obesity. Importantly, we observed no associations between RNI values and BMI or between puberty and RNI values among youths with HW-WS. Among youths with HW-WS, we interpret the positive association between age and RNI values as potentially indicating increased glial scavenger cell activity associated with synaptic pruning,^33^ although this requires histologic confirmation. After establishing reference associations, we observed that with varying weight gain, BMI had dissociable associations with RNI values beyond age and puberty. Across sexes, BMI was positively, independently, and bidirectionally associated with RNI, suggesting that BMI and RNI values are reciprocally related over time. We interpreted these associations to suggest that age and BMI have independent associations with RNI, reflecting that different causative mechanisms may exist for these associations.

Our results suggested sex specificity, with greater RNI values (possibly due to neuroinflammation) associated with greater weight gain in females. This finding may reflect differences in pubertal timing, which occurs earlier in females than in males.^58^ Here, males were largely prepubertal, as assessed on the pubertal development scale.^50^ Although greater RNI values in males was related to higher BMI, the inverse was also true, and these associations were largely stable. Therefore, it remains possible that the changes in RNI values related to weight gain may only become apparent at the onset of puberty. This is in line with our previous findings,^23^ showing that smaller subcortical volume was associated with weight gain in females, but not males. Importantly, despite this sex-specific association, our results with RNI values suggest that this metric may be more sensitive to detect associations between brain and weight in males than subcortical volume. Thus, changes in microstructure may occur prior to volumetric differences. Further work capturing the developmental phase as males continue to age and pass through puberty will be needed to better understand the association with microstructural variation. This will also be important to understand within the context of subcortical volume development, as indicated in our previously reported findings with females.^23^

In females in this study, higher RNI values corresponded with weight gain, possibly due to neuroinflammation. In animal models, neuroinflammation has been observed to occur in response to diet and even to precede weight gain.^29^ Dysregulation of appetite control via a neuroinflammatory mechanism may increase food intake, leading to compounded weight gain,^30^ which may also explain why smaller regionalized subcortical volumes were associated with greater weight gain.^23^ Many of the regions under investigation, including the amygdala, nucleus accumbens, hippocampus, pallidum, and thalamus, typically enlarge in volume size within this age range rather than undergo atrophic changes.^38^ Our finding of greater RNI values suggests that altered brain structure possibly due to neuroinflammation may represent a link between initial environment (including diet) associations on the brain and eventual weight gain in females. Future studies will be needed to address these specific mechanistic questions related to the association observed here.

### Strengths and Limitations

The study design provides insights into the associations among RNI, age, and BMI, providing a reference for how weight gain during adolescence may influence neurodevelopment. It also allowed us to disentangle the associations between RNI values and BMI and to quantify how RNI differences relate to unhealthy weight gain.

Study limitations include the lack of direct neuroinflammation measures (eg, astrogliosis or microglial activation) or neurodevelopment (eg, synaptic pruning), limiting a comprehensive mechanistic interpretation. There were no peripheral markers of systemic inflammation available (eg, C-reactive protein, interleukin-6, or tumor necrosis factor-α), limiting insight into whether RNI and BMI interacted with peripheral inflammation. Furthermore, it is unknown whether the individuals in the HW-NS group exhibited peripheral inflammation or if this classification accurately identifies unhealthy weight gain. The role of pubertal development in these associations remains unclear due to the problematic nature of self-reported answers on the pubertal development scale (without pictures of each Tanner stage) and salivary markers collected at inconsistent times of day. We used raw BMI as a marker for weight gain, which cannot provide insight into body composition or adiposity. Although age- and sex-specific BMI growth charts exist,^37^ they cannot track individual changes over time and are only useful for weight classification (eg, healthy weight, overweight, or obesity) by age and sex.^49^ Thus, raw BMI was preferred for our analyses and question of interest. The ABCD Study also lacked comprehensive assessment of variables that could provide additional insight into our questions, such as eating behavior and systemic inflammation.

## Conclusions

This cohort study sheds light on the associations between the natural progression of weight gain, neural cellularity, and development over a period of 2 years during early adolescence. RNI values were elevated during normal development, but excessive weight gain showed distinct associations with greater RNI values over time, and this was modulated by sex. Although we cannot determine whether these associations between greater weight gain and RNI values were attributable mechanistically to neuroinflammation or accelerated development, we found that in females, greater RNI values were associated with greater weight gain over time. This study provides additional support for the idea that neurobiological factors, such as potential neuroinflammation, may underlay and/or promote weight gain due to potential dysregulated appetitive control and its associated cognitive and metabolic consequences. Future mechanistic studies are needed to determine the specific cellular changes underlying these associations.

## Notes

Supplement 1.
eMethods

eResults

eDiscussion

eReferences
**eFigure 1.** Overview of the Number of Youths in Each Analysis **eFigure 2.** Associations for Males for the Analyses That Examined Whether an Arbitrary Threshold Cutoff Explained Differences in Restricted Normalized Isotropic (RNI) Values and Vice Versa **eFigure 3.** Associations for Females for the Analyses That Examined Whether an Arbitrary Threshold Cutoff Explained Differences in Restricted Normalized Isotropic (RNI) Values and Vice Versa **eFigure 4.** Associations Between Body Mass Index (BMI) and Age for Males and Females **eTable 1.** Exclusion Criteria **eTable 2.** Year 2 Demographics for All Youths Who Had a Healthy Weight at Baseline, Stratified by Sex **eTable 3.** Demographics for Males Classified as Healthy Weight, Weight Stable (HW-WS) and (Initially) Healthy Weight, Nonstable (HW-NS) **eTable 4.** Demographics for Females Classified as Healthy Weight, Weight Stable (HW-WS) and (Initially) Healthy Weight, Nonstable (HW-NS) **eTable 5.** Results From Model 1 in Males Who Were Classified as Healthy Weight, Weight Stable (HW-WS) **eTable 6.** Results From Model 2 in Males Who Were Classified as Healthy Weight, Weight Stable (HW-WS) **eTable 7.** Results for Model 1 Among Males With Various Amounts of Weight Gain (ie, Relatively Weight Stable to Relatively Unhealthy Weight Gain) **eTable 8.** Results for Model 2 Among Males With Various Amounts of Weight Gain (ie, Relatively Weight Stable to Relatively Unhealthy Weight Gain) **eTable 9.** Results for Model 1 for Females Who Were Classified as Healthy Weight, Weight Stable (HW-WS) **eTable 10.** Results for Model 2 for Females Who Were Classified as Healthy Weight, Weight Stable (HW-WS) **eTable 11.** Results for Model 1 for Females With Various Amounts of Weight Gain (ie, Relatively Weight Stable to Relatively Unhealthy Weight Gain) **eTable 12.** Results for Model 2 for Females With Various Amounts of Weight Gain (ie, Relatively Weight Stable to Relatively Unhealthy Weight Gain) **eTable 13.** Results From the Logistic Regression for Model 1 in Males at Baseline **eTable 14.** Results From the Logistic Regression for Model 1 in Males at Year 2 **eTable 15.** Results From the Logistic Regression for Model 2 in Males at Baseline **eTable 16.** Results From the Logistic Regression for Model 2 in Males at Year 2 **eTable 17.** Results From the Logistic Regression for Model 1 in Females at Baseline **eTable 18.** Results From the Logistic Regression for Model 1 in Females at Year 2 **eTable 19.** Results From the Logistic Regression for Model 2 in Females at Baseline **eTable 20.** Results From the Logistic Regression for Model 2 in Females at Year 2 **eTable 21.** Results for Model 1 for All Youths (Males and Females) Who Were Classified as Healthy Weight, Weight Stable (HW-WS) **eTable 22.** Results for Model 2 for All Youths (Males and Females) Classified as Healthy Weight, Weight Stable (HW-WS) **eTable 23.** Results for Model 1 for All Youths (Males and Females) With Various Amounts of Weight Gain (ie, Relatively Weight Stable to Relatively Unhealthy Weight Gain) **eTable 24.** Results for Model 2 for All Youths (Males and Females) With Various Amounts of Weight Gain (ie, Relatively Weight Stable to Relatively Unhealthy Weight Gain)

Supplement 2.
Data Sharing Statement

## References

[1] Sarma S, Sockalingam S, Dash S. Obesity as a multisystem disease: trends in obesity rates and obesity-related complications. Diabetes Obes Metab. 2021;23(suppl 1):3-16. doi:10.1111/dom.14290 33621415

[2] Gurnani M, Birken C, Hamilton J. Childhood obesity: causes, consequences, and management. Pediatr Clin North Am. 2015;62(4):821-840. doi:10.1016/j.pcl.2015.04.001 26210619

[3] Morys F, Dadar M, Dagher A. Association between midlife obesity and its metabolic consequences, cerebrovascular disease, and cognitive decline. J Clin Endocrinol Metab. 2021;106(10):e4260-e4274. doi:10.1210/clinem/dgab135 33677592 PMC8475210

[4] Morys F, Potvin O, Zeighami Y, ; Alzheimer’s Disease Neuroimaging Initiative. Obesity-associated neurodegeneration pattern mimics Alzheimer’s disease in an observational cohort study. J Alzheimers Dis. 2023;91(3):1059-1071. doi:10.3233/JAD-220535 36565111 PMC9912737

[5] Jurdak N, Lichtenstein AH, Kanarek RB. Diet-induced obesity and spatial cognition in young male rats. Nutr Neurosci. 2008;11(2):48-54. doi:10.1179/147683008X301333 18510803

[6] Spyridaki EC, Simos P, Avgoustinaki PD, . The association between obesity and fluid intelligence impairment is mediated by chronic low-grade inflammation. Br J Nutr. 2014;112(10):1724-1734. doi:10.1017/S0007114514002207 25315424

[7] Wahid RM, Samy W, El-Sayed SF. Cognitive impairment in obese rat model: role of glial cells. Int J Obes (Lond). 2021;45(10):2191-2196. doi:10.1038/s41366-021-00880-9 34140627

[8] Naderali EK, Ratcliffe SH, Dale MC. Obesity and Alzheimer’s disease: a link between body weight and cognitive function in old age. Am J Alzheimers Dis Other Demen. 2009;24(6):445-449. doi:10.1177/1533317509348208 19801534 PMC10846277

[9] Kandimalla R, Thirumala V, Reddy PH. Is Alzheimer’s disease a Type 3 Diabetes? A critical appraisal. Biochim Biophys Acta Mol Basis Dis. 2017;1863(5):1078-1089. doi:10.1016/j.bbadis.2016.08.018 27567931 PMC5344773

[10] Nederkoorn C, Braet C, Van Eijs Y, Tanghe A, Jansen A. Why obese children cannot resist food: the role of impulsivity. Eat Behav. 2006;7(4):315-322. doi:10.1016/j.eatbeh.2005.11.005 17056407

[11] Lavagnino L, Arnone D, Cao B, Soares JC, Selvaraj S. Inhibitory control in obesity and binge eating disorder: a systematic review and meta-analysis of neurocognitive and neuroimaging studies. Neurosci Biobehav Rev. 2016;68:714-726. doi:10.1016/j.neubiorev.2016.06.041 27381956

[12] Li N, Yolton K, Lanphear BP, Chen A, Kalkwarf HJ, Braun JM. Impact of early-life weight status on cognitive abilities in children. Obesity (Silver Spring). 2018;26(6):1088-1095. doi:10.1002/oby.22192 29797555 PMC5975980

[13] Li ZA, Ray MK, Gu Y, Barch DM, Hershey T. Weight indices, cognition, and mental health from childhood to early adolescence. JAMA Pediatr. 2024;178(8):830-833. doi:10.1001/jamapediatrics.2024.1379 38829657 PMC11148784

[14] de Groot CJ, van den Akker ELT, Rings EHHM, Delemarre-van de Waal HA, van der Grond J. Brain structure, executive function and appetitive traits in adolescent obesity. Pediatr Obes. 2017;12(4):e33-e36. doi:10.1111/ijpo.12149 27241878

[15] Li ZA, Samara A, Ray MK, . Childhood obesity is linked to putative neuroinflammation in brain white matter, hypothalamus, and striatum. Cereb Cortex Commun. 2023;4(2):tgad007. doi:10.1093/texcom/tgad007 37207193 PMC10191798

[16] Mestre ZL, Bischoff-Grethe A, Eichen DM, Wierenga CE, Strong D, Boutelle KN. Hippocampal atrophy and altered brain responses to pleasant tastes among obese compared with healthy weight children. Int J Obes (Lond). 2017;41(10):1496-1502. doi:10.1038/ijo.2017.130 28572588 PMC5626610

[17] Bruce AS, Holsen LM, Chambers RJ, . Obese children show hyperactivation to food pictures in brain networks linked to motivation, reward and cognitive control. Int J Obes (Lond). 2010;34(10):1494-1500. doi:10.1038/ijo.2010.84 20440296 PMC6800141

[18] Laurent JS, Watts R, Adise S, . Associations among body mass index, cortical thickness, and executive function in children. JAMA Pediatr. 2020;174(2):170-177. doi:10.1001/jamapediatrics.2019.4708 31816020 PMC6902097

[19] Adise S, Allgaier N, Laurent J, . Multimodal brain predictors of current weight and weight gain in children enrolled in the ABCD study ®. Dev Cogn Neurosci. 2021;49:100948. doi:10.1016/j.dcn.2021.100948 33862325 PMC8066422

[20] Rapuano KM, Berrian N, Baskin-Sommers A, . Longitudinal evidence of a vicious cycle between nucleus accumbens microstructure and childhood weight gain. J Adolesc Health. 2022;70(6):961-969. doi:10.1016/j.jadohealth.2022.01.002 35248457 PMC9133207

[21] Rapuano KM, Laurent JS, Hagler DJ Jr, . Nucleus accumbens cytoarchitecture predicts weight gain in children. Proc Natl Acad Sci U S A. 2020;117(43):26977-26984. doi:10.1073/pnas.2007918117 33046629 PMC7604478

[22] Adise S, Boutelle KN, Rezvan PH, . Sex-specific impulsivity, but not other facets of executive function, predicts fat and sugar intake two-years later amongst adolescents with a healthy weight: findings from the ABCD Study. Appetite. 2024;192:107081. doi:10.1016/j.appet.2023.107081 37839556 PMC10842015

[23] Adise S, Ottino-Gonzalez J, Hayati Rezvan P, . Smaller subcortical volume relates to greater weight gain in girls with initially healthy weight. Obesity (Silver Spring). 2024;32(7):1389-1400. doi:10.1002/oby.24028 38710591 PMC11211063

[24] Toschke AM, Rückinger S, Reinehr T, von Kries R. Growth around puberty as predictor of adult obesity. Eur J Clin Nutr. 2008;62(12):1405-1411. doi:10.1038/sj.ejcn.1602888 17717535

[25] Blakemore SJ, Burnett S, Dahl RE. The role of puberty in the developing adolescent brain. Hum Brain Mapp. 2010;31(6):926-933. doi:10.1002/hbm.21052 20496383 PMC3410522

[26] Miller AA, Spencer SJ. Obesity and neuroinflammation: a pathway to cognitive impairment. Brain Behav Immun. 2014;42:10-21. doi:10.1016/j.bbi.2014.04.001 24727365

[27] Leng F, Edison P. Neuroinflammation and microglial activation in Alzheimer disease: where do we go from here? Nat Rev Neurol. 2021;17(3):157-172. doi:10.1038/s41582-020-00435-y 33318676

[28] Li ZA, Cai Y, Taylor RL, . Associations between socioeconomic status, obesity, cognition, and white matter microstructure in children. JAMA Netw Open. 2023;6(6):e2320276. doi:10.1001/jamanetworkopen.2023.20276 37368403 PMC10300724

[29] Thaler JP, Yi CX, Schur EA, . Obesity is associated with hypothalamic injury in rodents and humans. J Clin Invest. 2012;122(1):153-162. doi:10.1172/JCI59660 22201683 PMC3248304

[30] Guillemot-Legris O, Muccioli GG. Obesity-induced neuroinflammation: beyond the hypothalamus. Trends Neurosci. 2017;40(4):237-253. doi:10.1016/j.tins.2017.02.005 28318543

[31] White NS, Leergaard TB, D’Arceuil H, Bjaalie JG, Dale AM. Probing tissue microstructure with restriction spectrum imaging: Histological and theoretical validation. Hum Brain Mapp. 2013;34(2):327-346. doi:10.1002/hbm.21454 23169482 PMC3538903

[32] Yang QQ, Zhou JW. Neuroinflammation in the central nervous system: symphony of glial cells. Glia. 2019;67(6):1017-1035. doi:10.1002/glia.23571 30548343

[33] Palmer CE, Pecheva D, Iversen JR, . Microstructural development from 9 to 14 years: evidence from the ABCD Study. Dev Cogn Neurosci. 2022;53:101044. doi:10.1016/j.dcn.2021.101044 34896850 PMC8671104

[34] Paolicelli RC, Bolasco G, Pagani F, . Synaptic pruning by microglia is necessary for normal brain development. Science. 2011;333(6048):1456-1458. doi:10.1126/science.1202529 21778362

[35] Dziabis JE, Bilbo SD. Microglia and sensitive periods in brain development. Curr Top Behav Neurosci. 2022;53:55-78. doi:10.1007/7854_2021_242 34463934

[36] Adolescent Brain Cognitive Development (ABCD) Study. Accessed June 13, 2025. https://abcdstudy.org/

[37] Kuczmarski RJ, Ogden CL, Guo SS, . 2000 CDC Growth Charts for the United States: methods and development. Vital Health Stat 11. 2002;(246):1-190. 12043359

[38] Herting MM, Johnson C, Mills KL, . Development of subcortical volumes across adolescence in males and females: a multisample study of longitudinal changes. Neuroimage. 2018;172:194-205. doi:10.1016/j.neuroimage.2018.01.020 29353072 PMC5910239

[39] Goddings AL, Mills KL, Clasen LS, Giedd JN, Viner RM, Blakemore SJ. The influence of puberty on subcortical brain development. Neuroimage. 2014;88:242-251. doi:10.1016/j.neuroimage.2013.09.073 24121203 PMC3991320

[40] Kung PH, Soriano-Mas C, Steward T. The influence of the subcortex and brain stem on overeating: how advances in functional neuroimaging can be applied to expand neurobiological models to beyond the cortex. Rev Endocr Metab Disord. 2022;23(4):719-731. doi:10.1007/s11154-022-09720-1 35380355 PMC9307542

[41] Davidson TL, Jones S, Roy M, Stevenson RJ. The cognitive control of eating and body weight: it’s more than what you “think”. Front Psychol. 2019;10(FEB):62. doi:10.3389/fpsyg.2019.00062 30814963 PMC6381074

[42] Nakamura Y, Ozawa S, Koike S. Caudate functional connectivity associated with weight change in adolescents. Front Hum Neurosci. 2020;14:587763. doi:10.3389/fnhum.2020.587763 33304257 PMC7701280

[43] Yokum S, Gearhardt AN, Harris JL, Brownell KD, Stice E. Individual differences in striatum activity to food commercials predict weight gain in adolescents. Obesity (Silver Spring). 2014;22(12):2544-2551. doi:10.1002/oby.20882 25155745 PMC4236252

[44] Casey BJ, Cannonier T, Conley MI, ; ABCD Imaging Acquisition Workgroup. The Adolescent Brain Cognitive Development (ABCD) Study: imaging acquisition across 21 sites. Dev Cogn Neurosci. 2018;32:43-54. doi:10.1016/j.dcn.2018.03.001 29567376 PMC5999559

[45] Garavan H, Bartsch H, Conway K, . Recruiting the ABCD sample: design considerations and procedures. Dev Cogn Neurosci. 2018;32(April):16-22. doi:10.1016/j.dcn.2018.04.004 29703560 PMC6314286

[46] Uban KA, Horton MK, Jacobus J, ; Adolescent Brain Cognitive Development Study. Biospecimens and the ABCD study: rationale, methods of collection, measurement and early data. Dev Cogn Neurosci. 2018;32(March):97-106. doi:10.1016/j.dcn.2018.03.005 29606560 PMC6487488

[47] Jernigan TL, Brown SA, Dowling GJ. The Adolescent Brain Cognitive Development Study. J Res Adolesc. 2018;28(1):154-156. doi:10.1111/jora.12374 29460352 PMC7477916

[48] Adolescent Brain Cognitive Development Study (ABCD) Annual Release 4.0 #634. National Institute of Mental Health Data Archive. Accessed June 13, 2025. https://nda.nih.gov/study.html?id=1299

[49] Adise S, Rhee KE, Laurent J, . Limitations of BMI z scores for assessing weight change: a clinical tool versus individual risk. Obesity (Silver Spring). 2024;32(3):445-449. doi:10.1002/oby.23957 38192094 PMC10922421

[50] Carskadon MA, Acebo C. A self-administered rating scale for pubertal development. J Adolesc Health. 1993;14(3):190-195. doi:10.1016/1054-139X(93)90004-9 8323929

[51] Hagler DJ Jr, Hatton S, Cornejo MD, . Image processing and analysis methods for the Adolescent Brain Cognitive Development Study. Neuroimage. 2019;202(August):116091. doi:10.1016/j.neuroimage.2019.116091 31415884 PMC6981278

[52] Samara A, Murphy T, Strain J, . Neuroinflammation and white matter alterations in obesity assessed by diffusion basis spectrum imaging. Front Hum Neurosci. 2020;13:464. doi:10.3389/fnhum.2019.00464 31992978 PMC6971102

[53] Samara A, Li Z, Rutlin J, . Nucleus accumbens microstructure mediates the relationship between obesity and eating behavior in adults. Obesity (Silver Spring). 2021;29(8):1328-1337. doi:10.1002/oby.23201 34227242 PMC8928440

[54] Wang Y, Sun P, Wang Q, . Differentiation and quantification of inflammation, demyelination and axon injury or loss in multiple sclerosis. Brain. 2015;138(Pt 5):1223-1238. doi:10.1093/brain/awv046 25724201 PMC4407189

[55] Cross AH, Song SK. “A new imaging modality to non-invasively assess multiple sclerosis pathology”. J Neuroimmunol. 2017;304:81-85. doi:10.1016/j.jneuroim.2016.10.002 27773433 PMC5316501

[56] Jolly E. Pymer4: connecting R and Python for linear mixed modeling. J Open Source Softw. 2018;3(31):862. doi:10.21105/joss.00862

[57] Jolly E. Pymer4: generalized linear and multi-level models in Python. 2025. Accessed June 13, 2025. https://github.com/ejolly/pymer4

[58] Bordini B, Rosenfield RL. Normal pubertal development: part II: clinical aspects of puberty. Pediatr Rev. 2011;32(7):281-292. doi:10.1542/pir.32.7.281 21724902

