# Supplementary material for: Distinct Patterns of Weight Gain, Age, and Subcortical Microstructure in Early Adolescence
Source: JAMA Netw Open. 2025 Jul 23;8(7):e2522211. doi: 10.1001/jamanetworkopen.2025.22211 (PMC12287857; doi:10.1001/jamanetworkopen.2025.22211)
Supplement: Supplement 1. — eMethods eResults eDiscussion eReferences eFigure 1. Overview of the Number of Youths in Each Analysis eFigure 2. Associations for Males for the Analyses That Examined Whether an Arbitrary Threshold Cutoff Explained Differences in Restricted Normalized Isotropic (RNI) Values and Vice Versa eFigure 3. Associations for Females for the Analyses That Examined Whether an Arbitrary Threshold Cutoff Explained Differences in Restricted Normalized Isotropic (RNI) Values and Vice Versa eFigure 4. Associations Between Body Mass Index (BMI) and Age for Males and Females eTable 1. Exclusion Criteria eTable 2. Year 2 Demographics for All Youths Who Had a Healthy Weight at Baseline, Stratified by Sex eTable 3. Demographics for Males Classified as Healthy Weight, Weight Stable (HW-WS) and (Initially) Healthy Weight, Nonstable (HW-NS) eTable 4. Demographics for Females Classified as Healthy Weight, Weight Stable (HW-WS) and (Initially) Healthy Weight, Nonstable (HW-NS) eTable 5. Results From Model 1 in Males Who Were Classified as Healthy Weight, Weight Stable (HW-WS) eTable 6. Results From Model 2 in Males Who Were Classified as Healthy Weight, Weight Stable (HW-WS) eTable 7. Results for Model 1 Among Males With Various Amounts of Weight Gain (ie, Relatively Weight Stable to Relatively Unhealthy Weight Gain) eTable 8. Results for Model 2 Among Males With Various Amounts of Weight Gain (ie, Relatively Weight Stable to Relatively Unhealthy Weight Gain) eTable 9. Results for Model 1 for Females Who Were Classified as Healthy Weight, Weight Stable (HW-WS) eTable 10. Results for Model 2 for Females Who Were Classified as Healthy Weight, Weight Stable (HW-WS) eTable 11. Results for Model 1 for Females With Various Amounts of Weight Gain (ie, Relatively Weight Stable to Relatively Unhealthy Weight Gain) eTable 12. Results for Model 2 for Females With Various Amounts of Weight Gain (ie, Relatively Weight Stable to Relatively Unhealthy Weight Gain) eTable 13. Results From the Logistic Regre [file jamanetwopen-e2522211-s001.pdf]

Supplemental Online Content

Adise S, Li ZA, Ottino-González J, Morys F, Chiarelli PA, Hershey T. Distinct patterns of weight gain, age, and subcortical microstructure in early adolescence. *JAMA Netw Open*. 2025;8(7):e2522211. doi:10.1001/jamanetworkopen.2025.22211

eMethods

eResults

eDiscussion

eReferences

eFigure 1. Overview of the Number of Youths in Each Analysis

eFigure 2. Associations for Males for the Analyses That Examined Whether an Arbitrary Threshold Cutoff Explained Differences in Restricted Normalized Isotropic (RNI) Values and Vice Versa

eFigure 3. Associations for Females for the Analyses That Examined Whether an Arbitrary Threshold Cutoff Explained Differences in Restricted Normalized Isotropic (RNI) Values and Vice Versa

eFigure 4. Associations Between Body Mass Index (BMI) and Age for Males and Females

eTable 1. Exclusion Criteria

eTable 2. Year 2 Demographics for All Youths Who Had a Healthy Weight at Baseline, Stratified by Sex

eTable 3. Demographics for Males Classified as Healthy Weight, Weight Stable (HW-WS) and (Initially) Healthy Weight, Nonstable (HW-NS)

eTable 4. Demographics for Females Classified as Healthy Weight, Weight Stable (HW-WS) and (Initially) Healthy Weight, Nonstable (HW-NS)

eTable 5. Results From Model 1 in Males Who Were Classified as Healthy Weight, Weight Stable (HW-WS)

eTable 6. Results From Model 2 in Males Who Were Classified as Healthy Weight, Weight Stable (HW-WS)

eTable 7. Results for Model 1 Among Males With Various Amounts of Weight Gain (ie, Relatively Weight Stable to Relatively Unhealthy Weight Gain)

eTable 8. Results for Model 2 Among Males With Various Amounts of Weight Gain (ie, Relatively Weight Stable to Relatively Unhealthy Weight Gain)

eTable 9. Results for Model 1 for Females Who Were Classified as Healthy Weight, Weight Stable (HW-WS)

© 2025 Adise S et al. *JAMA Network Open*.

eTable 10. Results for Model 2 for Females Who Were Classified as Healthy Weight, Weight Stable (HW-WS)

eTable 11. Results for Model 1 for Females With Various Amounts of Weight Gain (ie, Relatively Weight Stable to Relatively Unhealthy Weight Gain)

eTable 12. Results for Model 2 for Females With Various Amounts of Weight Gain (ie, Relatively Weight Stable to Relatively Unhealthy Weight Gain)

eTable 13. Results From the Logistic Regression for Model 1 in Males at Baseline

eTable 14. Results From the Logistic Regression for Model 1 in Males at Year 2

eTable 15. Results From the Logistic Regression for Model 2 in Males at Baseline

eTable 16. Results From the Logistic Regression for Model 2 in Males at Year 2

eTable 17. Results From the Logistic Regression for Model 1 in Females at Baseline

eTable 18. Results From the Logistic Regression for Model 1 in Females at Year 2

eTable 19. Results From the Logistic Regression for Model 2 in Females at Baseline

eTable 20. Results From the Logistic Regression for Model 2 in Females at Year 2

eTable 21. Results for Model 1 for All Youths (Males and Females) Who Were Classified as Healthy Weight, Weight Stable (HW-WS)

eTable 22. Results for Model 2 for All Youths (Males and Females) Classified as Healthy Weight, Weight Stable (HW-WS)

eTable 23. Results for Model 1 for All Youths (Males and Females) With Various Amounts of Weight Gain (ie, Relatively Weight Stable to Relatively Unhealthy Weight Gain)

eTable 24. Results for Model 2 for All Youths (Males and Females) With Various Amounts of Weight Gain (ie, Relatively Weight Stable to Relatively Unhealthy Weight Gain)

This supplemental material has been provided by the authors to give readers additional information about their work.

© 2025 Adise S et al. *JAMA Network Open*.

eMethods

Anthropometrics.

Participants were weighed (to the nearest 0.1lb) in light clothing and stocking feet using a physician's scale (Detecto model 439, Webb City, MO.) Height was measured on the same scale to the nearest 0.1 of an inch. Assessments occurred twice and were averaged, unless a large discrepancy occurred, in which case a third measurement was conducted. CDC's sex-age-height-weight specific BMI percentiles were used to categorize youth into weight class categories (e.g., healthy weight, overweight, obese),[1] but only raw BMI was utilized as a variable for the analyses.[2]

Pubertal assessment.

The Pubertal Development Scale[3] was administered to youth and the caregiver annually. Five sex-specific questions were answered on a 4-point scale: 1 = not yet started; 2 = barely started; 3 = definitely started; 4 = seems complete; 5 = I don't know (or missing). Tanner staging was calculated based on sex-specific responses (e.g., for females: body hair growth, breast development, menstruation; for males: body hair growth, voice changes, and facial hair growth). Tanner staging scores for males consisted of: Pre-pubescent = 3; early puberty = >3 and <6; mid puberty = >5 and <9; late puberty >8 and <12; post puberty ≥ 12. Tanner staging scores for females consisted of: pre- pubescent =3; early puberty =3 and no menarche; mid puberty = 4 and < 8 and no menarche; late puberty <8 and menarche; post puberty 8 and menarche. Participants who had missing values for any of the items needed to calculate Tanner staging were excluded.

Because not all caregivers are aware of the youth's pubertal growth, and the youth may overestimate, an average of the caregiver and youth Tanner stage (at each time point, respectively) was utilized. In rare cases, Tanner staging only existed for one responder (e.g., caregiver, youth), so no average existed as Tanner staging was based on only one report. Pubertal status was treated as a continuous variable, given it was possible to have values of 1.5 due to discrepancies in caregiver and youth reporting on the pubertal developmental scale (e.g., caregiver reported Tanner Stage 1 but youth reported Tanner Stage 2).

Kiddie Schedule for Affective Disorders and Schizophrenia (KSADS).

The KSADS[4] is a semi-structured interview that has an eating disorder module (e.g., bulimia nervosa, binge eating, anorexia nervosa). Diagnostic codes (e.g., 0=yes;1=no) were provided for past, present, or in remission. KSADS were administered to both the caregiver and youth. For the purposes of this manuscript, the KSADS was utilized for the purpose of exclusion from the analyses.

Race and Ethnicity.

At the baseline visit, the attending caregiver reported on the youth's race (16-options) and Ethnicity (2-options). This information was gathered for descriptive purposes to determine generalizability. In the current manuscript, race was collapsed into a 6-item variable (e.g., American Indian, Alaska Native/Native Hawaiian, Pacific Islander, Asian, Black, Mixed, Other,

© 2025 Adise S et al. *JAMA Network Open*.

White) for descriptive purposes only. The 16 response options were as follows: White, Black/African American, American Indian, Alaskan, Native Hawaiian, Guamanian, Samoan, Other Pacific Islander, Asian Indian, Chinese, Filipino, Japanese, Korean, Vietnamese, Other Asian, Other Race, and Mixed Race. Caregivers were also able to select: Refuse to Answer, and I don't know. Ethnicity was assessed as Hispanic/Latinx or Non-Hispanic/Non-Latinx.

Handedness.

Handedness (left, right, or ambidextrous) was assessed via the Youth Edinburgh Handedness Short Form.

Image acquisition and preprocessing.

Using 3T scanners (n=29), T1-weighted and multi-shell diffusion-weighted images were acquired. The ABCD Study Data Analytics Core preprocessed MRI images. Briefly, DWI data were corrected for distortion and head motion and registered to T1-weighted images. The RSI model was fitted to the DWI data, estimating voxel-based quantitative metrics within different tissue compartments. Estimates of restricted normalized isotropic (RNI) diffusion, which reflects water diffusion in cell bodies (e.g., glial cells, neurons) and is elevated in obesity and neuroinflammatory states, was included in the present analyses of subcortical brain tissue. RNI diffusion estimates for eight bilateral subcortical regions of interest (ROIs) were extracted using FreeSurfer's aseg atlas to garner estimates in the bilateral accumbens, amygdala, caudate, hippocampus, thalamus, pallidum, putamen, and ventral diencephalon (ventral DC).

Image quality control.

The ABCD Study Data Analytics Core performed basic quality control and provided guidance on exclusion metrics for data users to apply. As such, the following reasons resulted in DWI data not being included in the analyses: 1) the T1-weighted and DWI images were not usable; 2) DWI number of repetitions < 103; 3) failed FreeSurfer postprocessing; 4) B0 unwarp unavailable; 5) post processing failed; 6) registration to T1w > 17; 7) dorsal cutoff >47; 8) ventral cutoff >54; 9) derived results did not exist (quality control variable labeled: imgincl_dmri_include); and 10) mean framewise displacement >1.7 mm.

Exclusion Criteria.

Eligibility for the ABCD study was largely inclusive, but the following were reasons for exclusion at enrollment: MRI contraindications, history of major neurological disorders, prematurity at birth <28 weeks and/or hospitalization at birth >30 days, uncorrected vision, and known substance abuse problems. The current manuscript had additional exclusion criteria that were necessary to obtain an optimal sample for the hypotheses. Youth were excluded for the following reasons: (1) youth who took medications known to alter food intake (e.g., antipsychotics, insulin, stimulants); (2) caregiver report of neurological, psychiatric, or learning disabilities; (3) youth who met diagnostic criteria for eating disorders (e.g., anorexia, binge eating disorder) as assessed by the caregiver-reported KSADS;[5] (4) youth who were older than 12-years-old or had data collected after the COVID19 lockdown (e.g., 3/15/2020 - see note below); (5) Implausible anthropometrics (e.g., height [< 110 cm], or BMI values [> 100]); (6) decreasing height values (e.g., height at a follow-up visit less than previous of more than 2 cm);

© 2025 Adise S et al. *JAMA Network Open*.

(7) drastic increases in height values (e.g., height growth per year > 20 cm); (8) were underweight (according to the Center for Disease Control's (CDC's) age-sex-height-weight-specific growth curves)[1] as youth who were underweight or had lost weight were removed to avoid inclusion of those with potential restrictive eating or medical issues to make them underweight; (9) inconsistencies with sex-assigned at birth and/or gender identified on the sex-specific pubertal questionnaires; (10) missing demographic data; and 11) failed MRI quality control (e.g., poor image quality, movement). MRI quality control was performed by the ABCD Study's Data Analytics, Informatics, & Resource Center, but scoring criteria were provided for the user to apply. Of note, because ABCD often enrolled siblings, only one child per family (selected at random) was included in the analysis. Siblings were selected randomly, but after each exclusion criterion was applied in order to ensure our analysis included the maximum amount of data. Due to the COVID-19 pandemic, there was a pause in data collection, and only youth who had data collected prior to this period were included.

Exclusion of data due to COVID19 lockdown.

The COVID-19 lockdown in 2020 caused a pause in data collection during the year 2 follow-up. After lockdown restrictions were lifted, many families chose to conduct visits remotely; thus, height and weight were estimated. As this did not provide an accurate insight into weight gain, youth with estimated height and weight were excluded. There was a smaller subset of youth who had measured height and weight, but due to the inactivity of COVID and an increase in weight gain, these youth were also excluded from the analyses.

Data preparation.

Data were prepared using Python (v.3.12.15) and checked for implausible values, skewness, kurtosis, and multicollinearity using the variance inflation factor (*statsmodels,* v.0.14.2). Outliers were removed if values were 3 SD ± the mean. Continuous independent variables (e.g., puberty, intracranial volume, framewise displacement) were transformed into standardized scores using *scikit's* standard scaler package (v.1.4.2). Chi-squared (χ^2) and *t* tests were conducted to determine if groups differed among key demographics (see **Tables 1-2 and Tables S3-4**).

Linear mixed-effects models.

Linear mixed-effects models conducted in Python using the *pymer4* (v.0.8.2; https://github.com/ejolly/pymer4) examined the relationship between each ROI and RNI, age, and BMI over two years (see Model Framework in the main text). We used mixed models to manage missing data through full maximum likelihood estimation while addressing nested structures and examining population effects over time. Covariates were selected based on their known associations with BMI, puberty, highest caregiver education (effects coded), MRI motion (e.g., framewise displacement), and neurodevelopment (e.g., age). Time was modeled as age in months to account for developmental differences and the variation in time between study visits. Although age and puberty were highly correlated, variance inflation factor (VIF) analysis indicated little multicollinearity. We focused on age as the primary developmental variable, consistent with prior research showing that RNI changes are age-dependent. This decision was partly due to the accuracy limitations of Tanner-like staging and its reduced sensitivity to changes over time. Additionally, as Tanner-like staging does not capture underlying hormonal

changes, youth could remain in the same stage despite advancing in puberty, leading to potential ceiling effects over a two-year period. Given these concerns, we prioritized age over Tanner-like staging, as it may better capture both age-related and pubertal-related neurodevelopmental changes. Furthermore, due to known sex differences in maturation trajectories, possibly influenced by pubertal onset, we stratified analyses by sex. This approach accounts for sex-based variability in development while allowing age to serve as a general developmental proxy. Additional analyses tested for sex differences by inclusion of sex as an interaction term (see **Supplemental Materials eTables 21-24**). Income was not included as education is considered a more stable marker of socioeconomic status, and income was subject to a higher number of missing values. Finally, random effects were modeled for subject ID and MRI serial number (e.g., scanner ID). Crossed random (rather than nested random) effects were chosen given that it was possible that a youth was scanned on a different scanner at the same site. Although the MRI scanner manufacturer differed across sites, the scanner serial number captured both site-specific and scanner-related variance. Importantly, given that we had no hypotheses about race and ethnicity, nor was this manuscript focused on health disparities, these variables were not considered covariates. This is also in line with best practices in neuroimaging and nutrition research. To correct for multiple comparisons (n_{tests}=16), we applied the Benjamini-Hochberg false discovery rate method.

Model framework.

Grouping threshold: Finally, we explored whether a crude and arbitrary threshold to classify youth as HW-WS or HW-NS (i.e., not weight stable) could explain differences in RNI and vice versa. The impact of threshold choice for weight-group classification (e.g., HW-WS vs. HW-NS) was explored to determine if this grouping could serve as a clinically meaningful risk factor for RNI-related differences. Models 1 and 2 were implemented using logistic regression, with BMI replaced by the binary variable of group (HW-WS, HW-NS). As the grouping variable was time invariant, models were run at baseline and year 2.

© 2025 Adise S et al. *JAMA Network Open*.

eResults

Descriptive Statistics:

At baseline, 3,110 youth (49.7% females) were identified as having a healthy weight **(Table 1**), and 1,855 had adequate data at Y2 (**eTable 2**). Group classification (i.e., HW-WS vs. HW-NS) was dependent on having complete data at baseline and Y2. Accordingly, out of the 1,855 with complete data at Y2, 1,072 youth ($n_{females}$=480) met the HW-WS criterion and 773 youth (n_{males}=445) were classified as HW-NS (**Table 2,** demographic tables stratified by group and sex are provided in the **eTable 3-4**); 10 youth were excluded from the HW-WS group because they fluctuated between overweight to healthy weight between Y1 and Y2. On average, youth in the HW-WS group gained 7.6 ± 2.5 kg (over the two years), compared to 13.2 ± 3.9 kg (over the two years) of those in the HW-NS group. Notably, at Y2, just among those in the HW-NS group, 217 of the 773 youth (28.1%) transitioned to have overweight/obesity by the CDC cutoffs by Y2, while the remaining 556 youth (71.9%) remained in the healthy weight category, *even though they were not weight stable*. This suggests that utilizing the CDC weight classification cutoffs (e.g., healthy weight, overweight, obese) solely as markers for metabolic consequences may overlook unhealthy weight gain among youth in the healthy weight category. As such, it may be more insightful to examine within-person changes in BMI to determine weight stability.

Does group membership (i.e., HW-WS vs. HW-NS) predict RNI *in males*? No association was observed between group and RNI at either time point **(eTable 13-16, eFigure 2**). For Model 1, at baseline, age was associated with RNI across the subcortex, but there were no associations with puberty and RNI (**eTable 13).** By the year 2 time point, independent of age-RNI associations, puberty was associated with RNI in the bilateral accumbens, putamen, and left pallidum (p's < 0.05, uncorrected, **eTable 14**). However, results of model 2 showed that age and puberty were not associated with group membership (HW-WS vs. HW-NS) at either time point (**eTable 15-16**). Caregiver education was not included in the models due to issues with model convergence.

Does group membership (i.e., HW-WS vs. HW-NS) predict RNI *in females*: Results of Model 1 showed that at baseline group membership (HW-WS vs. HW-NS) was associated with differences in RNI in the bilateral accumbens, caudate, pallidum, and putamen, and left hippocampus (**eFigure 3,** Model 1, **eTable 17).** There were no associations with RNI and puberty at baseline. By the year 2 follow-up, group membership was associated with significant differences in the left hippocampus only (β= 0.002 (95% CI = [0.0, 0.003]), p=0.003). The bilateral accumbens, caudate, and putamen as well as the right hippocampus showed significant associations, but these did not pass multiple comparisons correction, potentially due to the observation that in these regions, puberty was also associated with RNI (**eTable 18**). Uncorrected results are only reported for exploratory purposes.

Results of Model 2 showed significant associations of age and puberty with group membership at both time points. At baseline, independent of this, RNI in the bilateral accumbens, caudate, and right putamen were associated with group membership, but these results did not survive multiple comparison testing (**eTable 19**). By the year 2 follow-up only the right caudate and left hippocampus were associated with group membership (**eTable 20**), but again, these associations did not survive multiple comparison testing and are only reported for exploratory purposes.

Does sex moderate the relationship between RNI and BMI among HW-WS youth: Results of Model 1 (outcome: RNI) showed that independent of BMI, age was significantly positively associated with RNI across the entire subcortex (all *p*'s < 0.05, FDR corrected). There were no significant three-way interactions between BMI*Age*Sex on RNI (**eTable 21**). Results of Model 2 (outcome: BMI) showed no three-way interactions between RNI*Age*Sex on BMI (**eTable 22**).

Does sex moderate the relationship between RNI and BMI among youth with variable weight gain: After established reference associations between RNI and BMI among HW-WS youth, we interrogated how these associations changed when including youth with various amounts of weight gain, including those who had transitioned to have overweight/obesity or were not weight stable but remained in the healthy weight category. Results from *Model 1* (outcome: RNI) showed no three-way interactions between BMI*Age*Sex (**eTable 23**). Results from *Model 2* (outcome: BMI) revealed three-way interactions between BMI*Age*Sex in the bilateral accumbens, hippocampus, putamen, and right caudate, left pallidum, and right thalamus (all *p's* < 0.05).

© 2025 Adise S et al. *JAMA Network Open*.

eDiscussion

We also examined whether the arbitrary cutoff applied to designate "unhealthy weight gain" was associated with differential relationships between RNI and BMI. This grouping variable was able to distinguish differences in RNI for females, but with less robust findings. In comparison to females with HW-WS, at baseline, those who were HW-NS exhibited significant differences in RNI in the bilateral accumbens, caudate, pallidum, and putamen, and left hippocampus (**eTable 17**). By the year 2 follow-up, group membership was only associated with differences in the left hippocampus (**eTable 18**). Underlying differences in RNI were not robust enough to correlate with group membership (Model 2). However, this binary classification showed some partial ability to distinguish group membership, although it did not mirror our results when BMI was assessed as a continuous variable. There are known drawbacks to utilizing a binary outcome in place of a continuous measure, as the variable may limit additional statistical significance gained by accounting for incremental effects on the outcome variable caused by the independent variable(s) in question. Therefore, the lack of robust associations with this grouping variable could be because it was too limiting, where the associations between RNI and BMI occur with larger amounts of weight gain.

Strengths and limitations:

The present study design benefited from the ability to describe the association between RNI, age, and BMI among HW-WS youth. As weight gain is hypothesized to affect neurodevelopment, understanding these relationships in the absence of excess weight serves as a reference point for disease progression. The study design also allowed us to disentangle the associations between RNI and BMI independent of age and quantify how differences in RNI relate to unhealthy weight gain.

Limitations of the current study include the lack of neuroimaging data in the study population that directly measures biological changes to neuroinflammation (e.g. astrogliosis, microglial activation) or neurodevelopment (e.g., synaptic pruning), providing limits for comprehensive mechanistic interpretation. There were no available peripheral markers of systemic inflammation (e.g., C-reactive protein, IL-6, TNF-alpha) in the ABCD Study, thus, not allowing for insight into whether RNI and BMI interacted with peripheral inflammation. Further, it is unknown if the individuals in the WG-NS group exhibited peripheral inflammation, and if this classification is, indeed, identifying unhealthy weight gain. Additionally, we are unable to infer the extent to which changes can be attributed to pubertal hormones. The ABCD Study collected self-reported answers to the pubertal development scale and salivary markers. However, there are known problems with both of these assessments. First, the pubertal development scale did not include pictures of each Tanner stage; therefore, interpretation of this data should be taken with caution. Second, although values of salivary markers of estrogen and testosterone exist, they were collected at different times of the day; for accuracy, these hormones must be collected in the morning within a short window after wakefulness. We utilized raw BMI as a marker for weight gain, and there are various caveats to this approach that have been detailed elsewhere. Additionally, raw BMI must be interpreted cautiously in children who are continuously growing, and while age and sex-specific growth charts exist, they cannot provide insight into changes over time. Nonetheless, BMI remains an imprecise assessment of body fat, and as such, more precise markers of adiposity would provide a clearer explanation of the relationships between RNI and

weight gain. The ABCD Study also lacked a comprehensive assessment of variables that could provide additional insight into our questions, such as eating behavior and systemic inflammation. Future studies are needed to carefully detail these associations.

© 2025 Adise S et al. *JAMA Network Open*.

eReferences

1. Kuczmarski, R. J., Ogden, C. L. & Guo, S. S. *2000 CDC Growth Charts for the United States: Methods and Development*. *National Center for Health Statistics. Vital Health Stat* vol. 11 (2002).
2. Adise, S. *et al.* Limitations of BMI *z* scores for assessing weight change: A clinical tool versus individual risk. *Obesity* **32**, 445–449 (2024).
3. Carskadon, M. A. & Acebo, C. A self-adminstered rating scale for pubertal development. *Journal of Adolescent Health* **14**, 190–195 (1993).
4. Kobak, K. A. & Kaufman, J. Ksads-comp. *Center for Telepsychology, Madison, WI* (2015).
5. Kaufman, J. *et al.* Schedule for affective disorders and schizophrenia for school-age children-present and lifetime version (K-SADS-PL): Initial reliability and validity data. *J Am Acad Child Adolesc Psychiatry* (1997) doi:10.1097/00004583-199707000-00021.

© 2025 Adise S et al. *JAMA Network Open*.

eFigure 1. Overview of the Number of Youths in Each Analysis

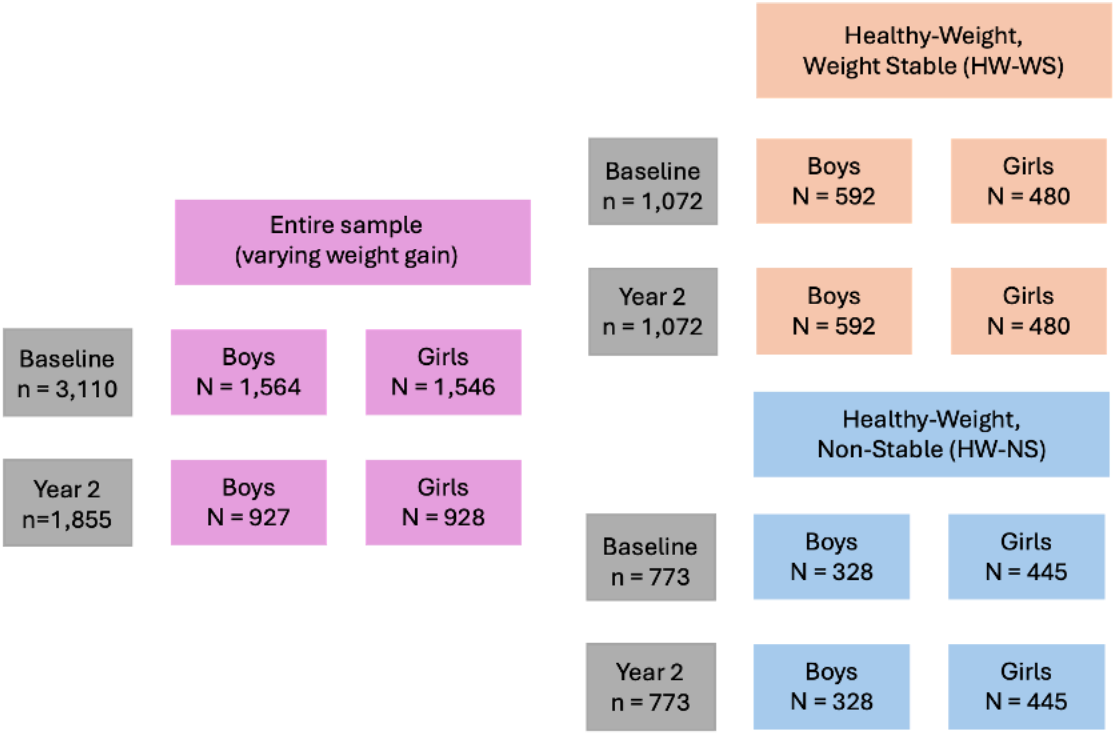

At baseline, there were 3,110 youth eligible for this study (i.e., all youth had a healthy weight). By year 2, data were available for only 1,855 youth. Among the 1,855 youth with available data, 1,072 were classified as healthy-weight, weight stable (HW-WS), while 773 youth were classified as healthy-weight, nonstable (HW-NS). Ten youth fluctuated from healthy weight to unhealthy weight to healthy weight across baseline through year 2, and as such did not meet the classification for either HW-WS or HW-NS. Purple corresponds to all youth (i.e., analyses across varying amounts of weight gain). Orange corresponds to the youth included in the HW-WS reference analyses. The HW-WS group was utilized to establish reference trajectories and, as a control for the group comparison in the logistic regression analysis, to determine if a grouping variable (e.g., HW-WS vs. HW-NS [blue boxes] could be a meaningful risk factor for RNI-related differences (results reported in **Supplemental Materials: Results, eFigure2-3, eTable13-20**).

© 2025 Adise S et al. *JAMA Network Open*.

eFigure 2. Associations for Males for the Analyses That Examined Whether an Arbitrary Threshold Cutoff Explained Differences in Restricted Normalized Isotropic (RNI) Values and Vice Versa

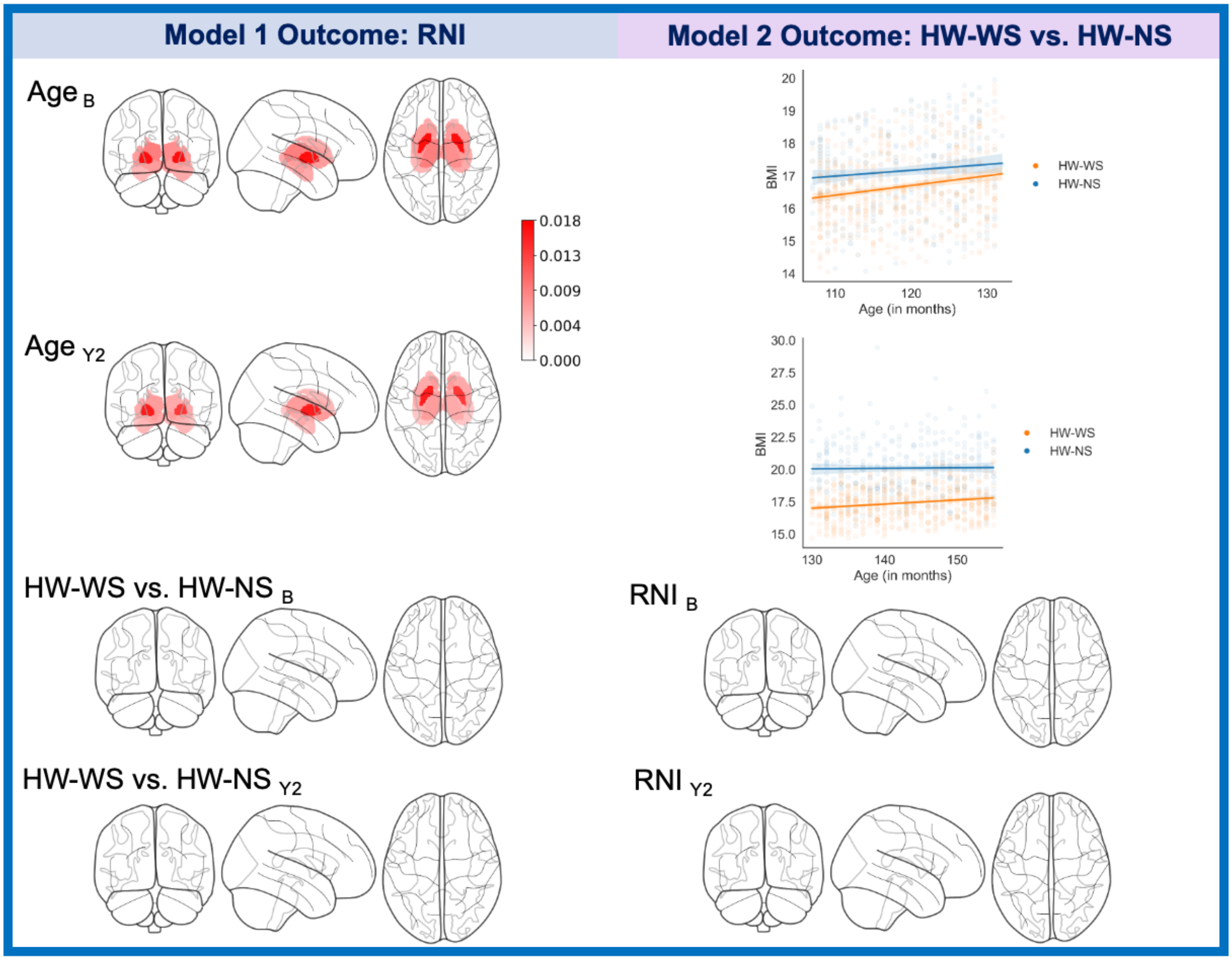

This threshold choice for group classification was explored to determine if this grouping could serve as a clinically meaningful risk factor for RNI-related differences. Model 1 shows the associations of whether group (e.g., healthy weight, weight stable [HW-WS] vs. healthy weight, nonstable [HW-NS]) was associated with differences in RNI. Model 2 shows the association of whether RNI was associated with group membership (e.g., HW-WS vs. HW-NS). Because group was time-invariant, models were run at baseline (B) and year (2) and results are reported at both time points for the associations of age on RNI, and age on BMI. A list of the significant effects for each model is displayed in **eTables 13-16.** The color bars reflect beta weights of the estimates for each region of interest.

© 2025 Adise S et al. *JAMA Network Open*.

eFigure 3. Associations for Females for the Analyses That Examined Whether an Arbitrary Threshold Cutoff Explained Differences in Restricted Normalized Isotropic (RNI) Values and Vice Versa

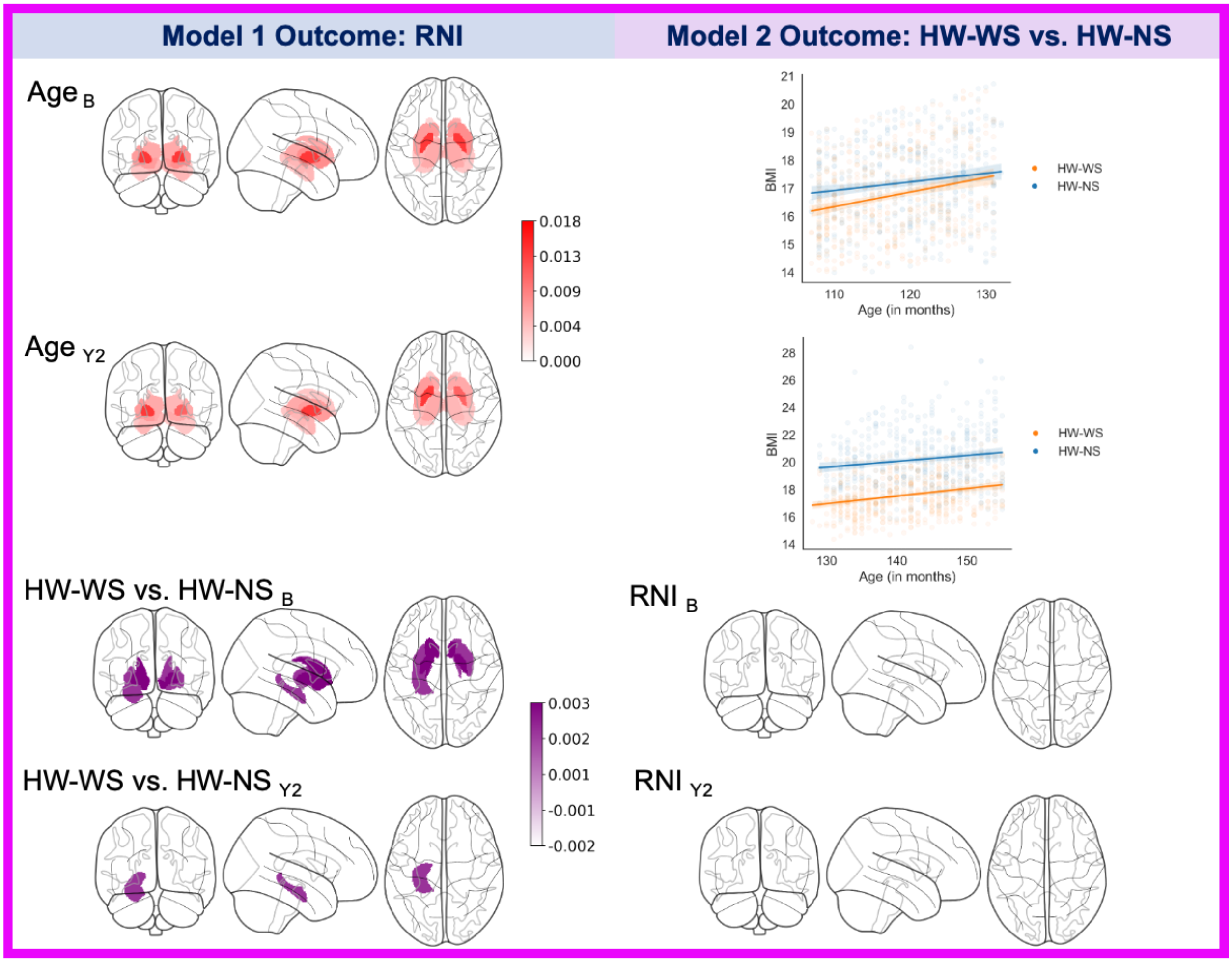

This threshold choice for group classification was explored to determine if this grouping could serve as a clinically meaningful risk factor for RNI-related differences. Model 1 shows the associations of whether group (e.g., healthy weight, weight stable [HW-WS] vs. healthy weight, nonstable [HW-NS]) was associated with differences in RNI. Model 2 shows the association of whether RNI was associated with group membership (e.g., HW-WS vs. HW-NS). Because group was time-invariant, models were run at baseline (B) and year (2) and results are reported at both time points for the associations of age on RNI, and age on BMI. A list of the significant effects for each model is displayed in **eTables 17-20.** The color bars reflect beta weights of the estimates for each region of interest.

© 2025 Adise S et al. *JAMA Network Open*.

eFigure 4. Associations Between Body Mass Index (BMI) and Age for Males and Females

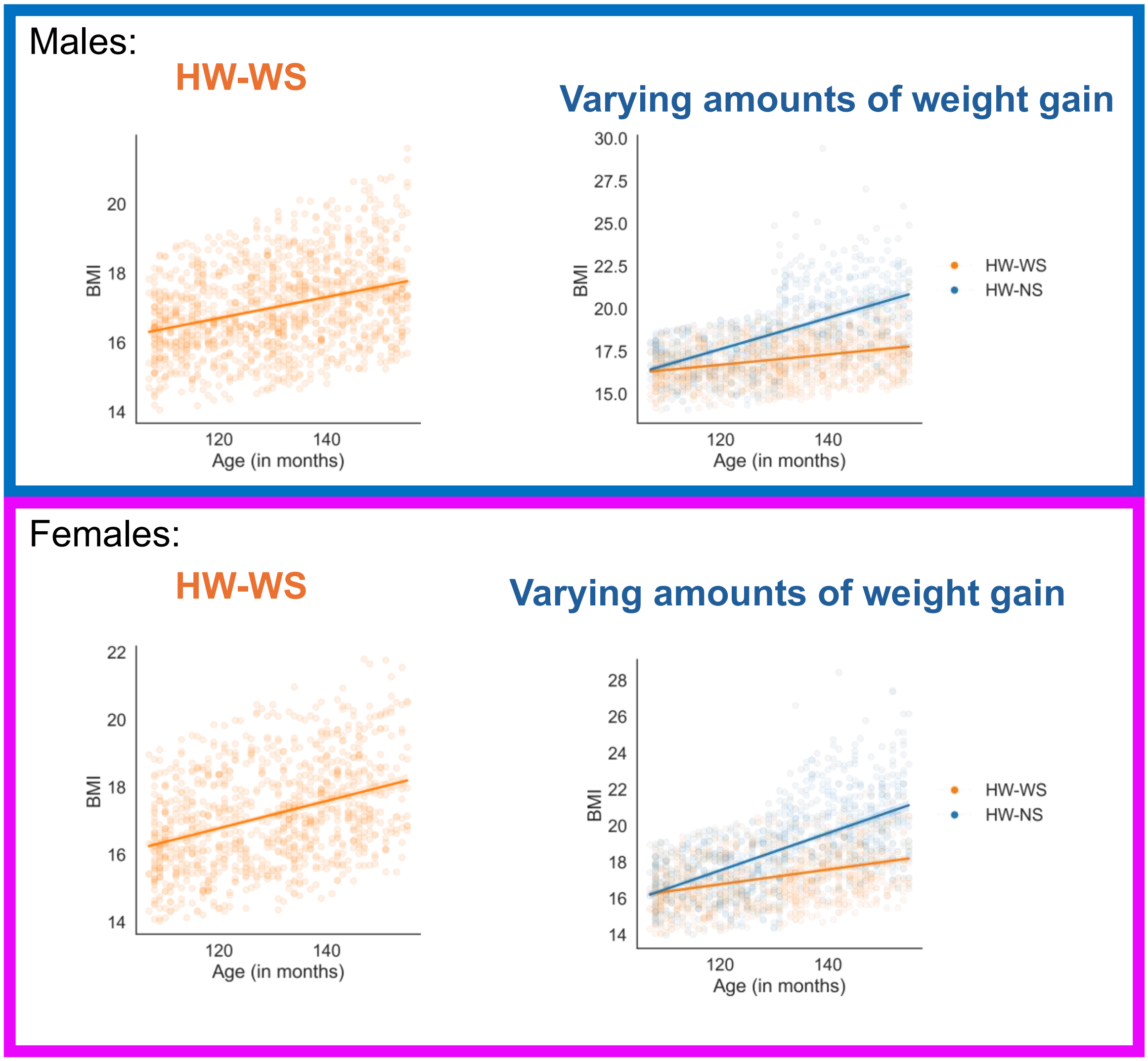

HW-WS = healthy weight, weight stable. HW-NS = healthy weight, nonstable

© 2025 Adise S et al. *JAMA Network Open*.

eTable 1. Exclusion Criteria

	n
Not taking medications know to affect food intake	11804
No learning disabilities or neurological disorders	11649
Did not meet criteria for eating disorders on the KSADS	11417
Data collected prior to COVID	11307
Age range within acceptable range	11295
Valid Anthropometric data	7775
No Missing Covariates	7588
Were of a healthy weight at baseline	4376
sMRI available at baseline	3892
Framewise displacement < 1.7	3485
Without siblings	3110
Year 2 data available	1855

Note. Metrics were generated starting with a data frame that consisted of available data for each category (e.g., BMI) at baseline or year 2 (Y2) but did not necessarily reflect the number of youths who had usable data at the baseline time point. KSADS = Kiddie Schedule for Affective Disorders and Schizophrenia. COVID = coronavirus-19. sMRI= structural magnetic resonance imaging. QC = quality control. Anthropometrics data were considered valid if the assessment was conducted in person, with no height discrepancies (e.g., assessment 2 shorter than the previous visit, implausible height growth [e.g., > 7 inches in a year], implausible height value [e.g., < 110 cm or more than 200 cm], BMI percentile < 5%, weight loss [due to potential restrictive eating], implausible BMI [e.g., BMI > 100], and missing height/weight variables). sMRI QC utilized the variable imgincl_dmri_include; Frame-wise displacement was derived from the variable iqc_dmri_ok_mean_motion.

© 2025 Adise S et al. *JAMA Network Open*.

eTable 2. Year 2 Demographics for All Youths Who Had a Healthy Weight at Baseline, Stratified by Sex

Variable		Missing	Overall	Females	Males	*p*
			1855	928	927	
**Sex, n (%)**	*Females*	--	928 (50.0)	928 (100.0)		
	Males	--	927 (50.0)		927 (100.0)	
**Age $_{Y2}$, mean (SD)**		--	142.4 (7.2)	142.2 (7.1)	142.6 (7.4)	0.17
**Puberty $_{Y2}$, mean (SD)**		--	2.6 (1.0)	3.3 (0.8)	1.9 (0.7)	**<0.001**
**BMI $_{Y2}$, mean (SD)**		--	18.6 (2.2)	18.8 (2.2)	18.4 (2.1)	**<0.001**
**Weight $_{Y2}$ (kg), mean (SD)**		--	43.0 (7.4)	43.9 (7.7)	42.0 (7.0)	**<0.001**
**Weight gain (kg), mean (SD)**		--	9.9 (4.2)	10.7 (4.2)	9.1 (4.0)	**<0.001**
**Education, n (%)**	*<HS*	--	42 (2.3)	23 (2.5)	19 (2.0)	0.46
	HS/GED	--	99 (5.3)	45 (4.8)	54 (5.8)	
	Some College	--	389 (21.0)	196 (21.1)	193 (20.8)	
	BA degree	--	532 (28.7)	280 (30.2)	252 (27.2)	
	Postgrad	--	793 (42.8)	384 (41.5)	409 (44.1)	
**Weight Class $_{B}$, n (%)**	*Healthy Weight*	--	1855 (100)	928 (100)	927 (100)	1.0
**Weight Class $_{Y2}$, n (%)**	*Healthy Weight*	--	1638 (88.3)	821 (88.5)	817 (88.1)	0.61
	Overweight	--	201 (10.8)	101 (10.8)	100 (10.8)	
	Obese	--	16 (0.9)	6 (0.6)	10 (1.1)	
**INR, mean (SD)**		125	4.2 (2.4)	4.1 (2.4)	4.2 (2.3)	0.60
**Race, n (%)**	*AIAN/NHPI*	--	8 (0.4)	7 (0.8)	1 (0.1)	0.18
	Asian	--	40 (2.2)	26 (2.8)	14 (1.5)	
	Black	--	163 (8.8)	80 (8.5)	83 (9.0)	
	Mixed	--	171 (9.2)	85 (9.21)	86 (9.3)	
	Other	--	64 (3.5)	34 (3.7)	30 (3.2)	
	White	--	1393 (75.1)	689 (74.2)	704 (75.9)	
	Missing	--	16 (0.9)	7 (0.8)	9 (1.0)	
**Ethnicity, n (%)**	*Latinx*	--	292 (15.8)	151 (16.3)	141 (15.2)	0.57
	Non-Latinx	--	1540 (83.0)	767 (82.7)	773 (83.4)	
	Missing	--	23 (1.2)	10 (1.1)	13 (1.4)	
**Income, n (%)**	*<$50k*	--	343 (18.5)	186 (20.0)	157 (16.9)	0.16
	$50-100k	--	527 (28.3)	270 (29.1)	257 (27.7)	
	>$100k	--	872 (47.1)	423 (45.6)	449 (48.4)	
	Missing	--	113 (6.1)	49 (5.3)	64 (6.9)	
**Household poverty rate, mean (SD)**		21	27682.0 (5472.3)	27478.5 (5400.4)	27885.1 (5538.7)	0.11

Note. Race (16 items, collapsed into 6 groups) and Ethnicity (2 items) were self-reported by the caregiver. AIAN/NHPI = American Indian, Alaska Native/Native Hawaiian, Pacific Islander; HS = High school; GED = Generalized Education Degree; BA = bachelor's degree; BMI = Body Mass Index; INR = Income to Needs Ratio. B = baseline; Y2 = year 2. Descriptive statistics are provided for self-reported race only for sample interpretation diversity. P-values reflect chi-squared and t-tests where appropriate. Data are mean (SD) or frequency and percentage (%). p<0.05 (bold font) for chi-squared and t-tests were appropriate. Weight class was determined using the 2000 CDC growth charts.

© 2025 Adise S et al. *JAMA Network Open*.

eTable 3. Demographics for Males Classified as Healthy Weight, Weight Stable (HW-WS) and (Initially) Healthy Weight, Nonstable (HW-NS)

Variable	Missing	HW-WS (n=592)	HW-NS (n=328)	*p*
		No (%)	No (%)	
**Education**				
<HS	--	9 (1.5)	10 (3.0)	**0.003**
HS/GED	--	26 (4.4)	28 (8.5)	
Some College	--	113 (19.1)	80 (24.4)	
BA degree	--	175 (29.6)	75 (22.9)	
Postgrad	--	269 (45.4)	135 (41.2)	
**Income**				
<$50k	--	154 (26.0)	100 (30.5)	0.20
$50-100k	--	95 (16.0)	62 (18.9)	
>$100k	--	299 (50.5)	146 (44.5)	
Missing	--	44 (7.4)	20 (6.1)	
**Race**				
AIAN/NHPI	--		1 (0.3)	0.1
Asian	--	8 (1.4)	6 (1.8)	
Black	--	45 (7.7)	38 (11.7)	
Mixed	--	60 (10.3)	25 (7.7)	
White	--	456 (77.9)	242 (74.2)	
Other	--	16 (2.7)	14 (4.3)	
Missing	9	7 (1.2)	2 (0.6)	
**Ethnicity**				
Latinx	--	76 (12.8)	63 (19.2)	**0.01**
Non-Latinx	--	516 (87.2)	265 (80.8)	
**Weight Class**				
Healthy Weight $_B$	--	592 (100.0)	328 (100.0)	>0.99
Healthy Weight $_{Y2}$	--		218 (66.5)	**<0.001**
Overweight $_{Y2}$	--		100 (30.5)	
Obese $_{Y2}$	--		10 (3.0)	
**Age (in months), mean (SD)**				
Baseline	--	118.6 (7.3)	118.9 (7.4)	0.61
Year 2	--	142.5 (7.4)	142.9 (7.4)	0.47
**Puberty, mean (SD)**				
Baseline	--	1.6 (0.5)	1.6 (0.5)	0.42
Year 2	--	1.9 (0.6)	2.0 (0.7)	**0.04**
**BMI, mean (SD)**				
Baseline	--	16.7 (1.2)	17.2 (1.3)	**<0.001**
Year 2	--	17.4 (1.3)	20.1 (2.1)	**<0.001**
**Weight (kg), mean (SD)**				
Baseline	--	32.4 (4.0)	33.7 (4.7)	**<0.001**
Year 2	--	39.5 (5.4)	46.4 (7.4)	
**Weight gain (kg), mean (SD)**	--	7.1 (2.4)	12.7 (4.0)	**<0.001**
**INR, mean (SD)**	70	4.3 (2.4)	3.9 (2.3)	**0.02**
**Household poverty rate, mean (SD)**	9	27895.7 (5201.1)	27833.5 (6018.4)	0.88

Note. Race (16 items, collapsed into 6 groups) and Ethnicity (2 items) were self-reported by the caregiver. AIAN/NHPI = American Indian, Alaska Native/Native Hawaiian, Pacific Islander; HS = High school; GED = Generalized Education Degree; BA = bachelor's degree; BMI = Body Mass Index; INR = Income to Needs Ratio. HW-WS= healthy weight, weight stable; HW-NS = (initially) healthy weight, nonstable. B = baseline; Y2 = year 2Descriptive statistics are reported for the interpretation of sample diversity. P-values reflect chi-squared and t-tests where appropriate. p<0.05 (bold font) for chi-squared and t-tests were appropriate. Weight class was determined using the 2000 CDC growth charts. Seven males that had data available at baseline and year 2 were not included in in the HW-WS category because they had fluctuated between weight class categories (e.g., healthy weight to unhealthy weight to healthy weight).

eTable 4. Demographics for Females Classified as Healthy Weight, Weight Stable (HW-WS) and (Initially) Healthy Weight, Nonstable (HW-NS)

Variable	Missing	HW-WS (n=480)	HW-NS (n=445)	*p*
		No (%)	No (%)	
**Education, n (%)**				
<HS	--	12 (2.5)	11 (2.5)	0.05
HS/GED	--	16 (3.3)	29 (6.5)	
Some College	--	89 (18.6)	106 (23.8)	
BA degree	--	151 (31.5)	127 (28.7)	
Postgrad	--	212 (44.2)	172 (38.7)	
**Income, n (%)**				
<$50k	--	134 (28.0)	133 (30.1)	**<0.001**
$50-100k	--	78 (16.3)	106 (24.0)	
>$100k	--	249 (52.0)	172 (38.9)	
Missing	--	18 (3.8)	31 (7.0)	
**Race, n (%)**				
AIAN/NHPI		5 (1.0)	2 (0.5)	0.18
Asian	--	15 (3.1)	11 (2.5)	
Black	--	32 (6.7)	47(10.6)	
Mixed	--	47 (9.8)	38 (8.5)	
White	--	365 (76.0)	322 (72.4)	
Other	--	14 (2.9)	20 (4.5)	
Missing	7	2 (0.4)	5 (1.1)	
**Ethnicity, n (%)**				
Latinx	--	66 (13.8)	84 (18.9)	**0.05**
Non-Latinx	--	414 (86.2)	361 (81.1)	
**Weight Class, n (%)**				
Healthy Weight $_{B}$	--	480 (100.0)	445 (100.0)	1.000
Healthy Weight $_{Y2}$	--		338 (76.0)	**<0.001**
Overweight $_{Y2}$	--		101 (22.7)	
Obese $_{Y2}$	--		6 (1.3)	
**Age, mean (SD)**				
Baseline	--	117.3 (6.7)	119.5 (7.1)	**<0.001**
Year 2	--	141.1 (6.8)	143.3 (7.2)	**<0.001**
**Puberty, mean (SD)**				
Baseline	--	1.9 (0.9)	2.3 (0.9)	**<0.001**
Year 2	--	3.1 (0.8)	3.5 (0.7)	**<0.001**
**BMI, mean (SD)**				
Baseline	--	16.7 (1.4)	17.2 (1.5)	**<0.001**
Year 2	--	17.6 (1.5)	20.2 (2.1)	**<0.001**
**Weight (kg), mean (SD)**				
Baseline	--	32.1 (4.8)	34.3 (5.3)	**<0.001**
Year 2	--	40.2 (5.9)	47.8 (7.5)	
**Weight gain (kg), mean (SD)**	--	8.1 (2.5)	13.5 (3.9)	**<0.001**
**INR, mean (SD)**	55	32.1 (4.8)	34.3 (5.3)	**<0.001**
**Household Poverty Rate, mean (SD)**	12	27298.7 (5156.1)	27640.5 (5654.9)	0.36

Note. Race (16 items, collapsed into 6 groups) and Ethnicity (2 items) were self-reported by the caregiver. AIAN/NHPI = American Indian, Alaska Native/Native Hawaiian, Pacific Islander; HS = High school; GED = Generalized Education Degree; BA = bachelor's degree; BMI = Body Mass Index; INR = Income to Needs Ratio. HW-WS= healthy weight, weight stable; HW-NS = (initially) healthy weight, nonstable. Descriptive statistics are reported for self-reported race only for the interpretation of sample diversity. P-values reflect chi-squared and t-tests where appropriate. Data are mean (SD) or frequency and percentage (%). $p<0.05$ (bold font) for chi-squared and t-tests were appropriate. Weight class was determined using the 2000 CDC growth charts. Three females that had data available at baseline and year 2 were not included in in the HW-WS category because they had fluctuated between weight class categories (e.g., healthy weight to unhealthy weight to healthy weight).

© 2025 Adise S et al. *JAMA Network Open*.

eTable 5. Results From Model 1 in Males Who Were Classified as Healthy Weight, Weight Stable (HW-WS)

RNI ~ BMI*Age + Puberty + Education + Motion + (1|MRI scanner ID) + (1|subject ID)

		Age		BMI		BMI*Age		Puberty	
**ROI**	**H**	**β (95% CI)**	***p***	**β (95% CI)**	***p***	**β (95% CI)**	***p***	**β (95% CI)**	***p***
Accumbens	L	0.006 (0.005, 0.007)	**<0.001**	0.001 (-0.001, 0.002)	0.47	-0.001 (-0.002, 0.0)	0.21	0.001 (-0.0, 0.003)	0.08
	R	0.006 (0.005, 0.007)	**<0.001**	0.0 (-0.001, 0.002)	0.68	-0.0 (-0.001, 0.001)	0.50	0.001 (-0.001, 0.002)	0.26
Amygdala	L	0.005 (0.004, 0.006)	**<0.001**	0.0 (-0.001, 0.002)	0.54	-0.0 (-0.001, 0.001)	0.58	-0.001 (-0.002, 0.001)	0.35
	R	0.005 (0.004, 0.006)	**<0.001**	0.001 (-0.001, 0.002)	0.38	-0.0 (-0.001, 0.001)	0.34	-0.001 (-0.002, 0.001)	0.29
Caudate	L	0.004 (0.003, 0.005)	**<0.001**	0.001 (-0.0, 0.003)	0.08	-0.001 (-0.002, 0.0)	0.19	0.001 (-0.001, 0.002)	0.26
	R	0.004 (0.003, 0.004)	**<0.001**	0.001 (-0.001, 0.002)	0.37	-0.001 (-0.002, 0.0)	**0.05**	0.0 (-0.001, 0.001)	0.83
Hippocampus	L	0.003 (0.003, 0.004)	**<0.001**	0.001 (-0.001, 0.002)	0.44	-0.0 (-0.001, 0.0)	0.24	0.0 (-0.001, 0.001)	0.94
	R	0.004 (0.003, 0.004)	**<0.001**	0.001 (-0.0, 0.002)	0.14	-0.001 (-0.002, -0.0)	**0.01**	-0.0 (-0.001, 0.001)	0.59
Pallidum	L	0.015 (0.013, 0.016)	**<0.001**	0.002 (-0.001, 0.005)	0.16	0.0 (-0.001, 0.002)	0.60	-0.002 (-0.004, 0.0)	0.13
	R	0.014 (0.013, 0.016)	**<0.001**	0.002 (-0.001, 0.004)	0.20	0.0 (-0.002, 0.002)	1.00	-0.001 (-0.003, 0.001)	0.35
Putamen	L	0.006 (0.005, 0.006)	**<0.001**	0.0 (-0.001, 0.002)	0.59	-0.0 (-0.001, 0.001)	0.89	0.0 (-0.001, 0.001)	0.93
	R	0.006 (0.005, 0.006)	**<0.001**	0.001 (-0.001, 0.002)	0.32	-0.0 (-0.001, 0.001)	0.78	0.001 (-0.0, 0.002)	0.30
Thalamus	L	0.006 (0.005, 0.007)	**<0.001**	0.001 (-0.001, 0.003)	0.39	-0.001 (-0.002, 0.0)	0.07	0.001 (-0.001, 0.002)	0.35
	R	0.006 (0.005, 0.007)	**<0.001**	0.0 (-0.001, 0.002)	0.69	-0.001 (-0.002, 0.0)	0.13	0.0 (-0.001, 0.002)	0.74
Ventral DC	L	0.006 (0.005, 0.007)	**<0.001**	0.001 (-0.0, 0.003)	0.12	-0.0 (-0.001, 0.001)	0.77	-0.0 (-0.002, 0.001)	0.66
	R	0.005 (0.004, 0.006)	**<0.001**	0.001 (-0.0, 0.003)	0.14	-0.001 (-0.002, 0.0)	0.08	-0.001 (-0.002, 0.0)	0.18

Note. Estimated regression coefficients (β), 95% confidence intervals (CI), and *p*-values from the linear mixed-effects models examining whether changes in BMI over time (i.e., age) led to changes in RNI. Models were controlled for the caregiver's highest education (effects coded) and framewise displacement. Random effects were modeled from subject ID and scanner serial number (e.g., MRI scanner ID). Significant effects are bolded, where * indicates that the association survived the Benjamini-Hochberg multiple comparison testing correction. BMI = body mass index. ROI=Region-of-interest; H=Hemisphere; L=Left; R=Right.

© 2025 Adise S et al. *JAMA Network Open*.

eTable 6. Results From Model 2 in Males Who Were Classified as Healthy Weight, Weight Stable (HW-WS)

BMI ~ RNI*Age + Puberty + Education + Motion + (1|MRI scanner ID) + (1|subject ID)

		**Age**		**RNI**		**RNI*Age**		**Puberty**	
**ROI**	**H**	**β (95% CI)**	***p***	**β (95% CI)**	***p***	**β (95% CI)**	***p***	**β (95% CI)**	***p***
Accumbens	L	0.38 (0.34, 0.42)	**<0.001**	0.02 (-0.04, 0.08)	0.49	-0.01 (-0.05, 0.02)	0.57	0.1 (0.02, 0.17)	**0.009**
	R	0.39 (0.35, 0.43)	**<0.001**	0.01 (-0.05, 0.07)	0.77	-0.01 (-0.04, 0.02)	0.57	0.09 (0.02, 0.16)	**0.01**
Amygdala	L	0.38 (0.34, 0.42)	**<0.001**	0.04 (-0.01, 0.1)	0.14	-0.0 (-0.04, 0.03)	0.85	0.11 (0.03, 0.18)	**0.004**
	R	0.38 (0.34, 0.42)	**<0.001**	0.04 (-0.02, 0.09)	0.21	-0.0 (-0.04, 0.03)	0.95	0.1 (0.02, 0.17)	**0.008**
Caudate	L	0.38 (0.34, 0.42)	**<0.001**	0.03 (-0.03, 0.08)	0.30	-0.02 (-0.05, 0.02)	0.35	0.1 (0.02, 0.17)	**0.008**
	R	0.39 (0.35, 0.43)	**<0.001**	-0.01 (-0.06, 0.05)	0.83	-0.02 (-0.06, 0.01)	0.16	0.1 (0.03, 0.17)	**0.007**
Hippocampus	L	0.38 (0.34, 0.42)	**<0.001**	0.03 (-0.02, 0.09)	0.26	0.01 (-0.03, 0.04)	0.74	0.09 (0.02, 0.17)	**0.01**
	R	0.37 (0.33, 0.42)	**<0.001**	0.05 (-0.01, 0.12)	0.10	-0.0 (-0.04, 0.03)	0.82	0.1 (0.03, 0.17)	**0.007**
Pallidum	L	0.36 (0.32, 0.41)	**<0.001**	0.07 (0.0, 0.14)	0.05	-0.01 (-0.04, 0.02)	0.63	0.11 (0.03, 0.18)	**0.004**
	R	0.37 (0.32, 0.41)	**<0.001**	0.05 (-0.02, 0.12)	0.15	-0.02 (-0.05, 0.02)	0.38	0.11 (0.04, 0.18)	**0.003**
Putamen	L	0.38 (0.34, 0.43)	**<0.001**	0.02 (-0.05, 0.09)	0.58	-0.01 (-0.04, 0.02)	0.54	0.1 (0.03, 0.17)	**0.008**
	R	0.37 (0.33, 0.42)	**<0.001**	0.04 (-0.03, 0.1)	0.29	-0.02 (-0.05, 0.02)	0.31	0.1 (0.02, 0.17)	**0.008**
Thalamus	L	0.38 (0.34, 0.42)	**<0.001**	0.02 (-0.04, 0.09)	0.45	-0.01 (-0.05, 0.02)	0.46	0.1 (0.03, 0.17)	**0.007**
	R	0.38 (0.34, 0.42)	**<0.001**	0.02 (-0.04, 0.09)	0.52	-0.01 (-0.04, 0.03)	0.64	0.1 (0.03, 0.17)	**0.006**
Ventral DC	L	0.37 (0.33, 0.41)	**<0.001**	0.07 (0.0, 0.13)	0.03	0.0 (-0.03, 0.04)	0.89	0.1 (0.03, 0.17)	**0.007**
	R	0.38 (0.34, 0.42)	**<0.001**	0.06 (-0.01, 0.12)	0.09	0.0 (-0.03, 0.04)	0.80	0.1 (0.03, 0.17)	**0.008**

Note. Estimated regression coefficients (β), 95% confidence intervals (CI), and *p*-values from the linear mixed-effects models examining whether changes in RNI over time (i.e., age) led to changes in BMI. Models were controlled for the caregiver's highest education (effects coded) and framewise displacement. A random effect was modeled for subject, while MRI scanner ID was not modeled due to singularity issues. Significant effects are bolded, where * indicates that the association survived the Benjamini-Hochberg multiple comparison testing correction. BMI = body mass index. ROI=Region-of-interest; H=Hemisphere; L=Left; R=Right.

© 2025 Adise S et al. *JAMA Network Open*.

eTable 7. Results for Model 1 Among Males With Various Amounts of Weight Gain (ie, Relatively Weight Stable to Relatively Unhealthy Weight Gain)

RNI ~ BMI*Age + Puberty + Education + Motion + (1|MRI scanner ID) + (1|subject ID)

ROI	H	Age β (95% CI)	Age *p*	BMI β (95% CI)	BMI *p*	BMI*Age β (95% CI)	BMI*Age *p*	Puberty β (95% CI)	Puberty *p*
Accumbens	L	0.005 (0.005, 0.006)	**<0.001**	0.002 (0.001, 0.003)	**<0.001***	-0.0 (-0.001, 0.0)	0.49	0.001 (0.001, 0.002)	**0.003**
	R	0.005 (0.005, 0.006)	**<0.001**	0.002 (0.001, 0.002)	**<0.001***	-0.0 (-0.001, 0.001)	0.78	0.001 (0.0, 0.002)	**0.009**
Amygdala	L	0.005 (0.004, 0.005)	**<0.001**	0.001 (-0.0, 0.001)	0.20	-0.0 (-0.001, 0.0)	0.19	-0.0 (-0.001, 0.001)	0.61
	R	0.005 (0.004, 0.005)	**<0.001**	0.001 (0.0, 0.002)	**0.03***	-0.0 (-0.001, 0.0)	0.27	-0.0 (-0.001, 0.001)	0.66
Caudate	L	0.004 (0.004, 0.005)	**<0.001**	0.001 (0.0, 0.002)	**0.01***	-0.0 (-0.001, 0.0)	0.31	0.001 (-0.0, 0.002)	0.11
	R	0.003 (0.003, 0.004)	**<0.001**	0.001 (0.001, 0.002)	**0.001***	-0.001 (-0.001, 0.0)	0.06	0.001 (-0.0, 0.001)	0.19
Hippocampus	L	0.003 (0.003, 0.004)	**<0.001**	0.001 (-0.0, 0.001)	0.11	-0.0 (-0.001, 0.0)	0.17	0.0 (-0.001, 0.001)	0.85
	R	0.004 (0.003, 0.004)	**<0.001**	0.001 (-0.0, 0.001)	**0.05**	-0.0 (-0.001, -0.0)	**0.04**	-0.0 (-0.001, 0.0)	0.46
Pallidum	L	0.014 (0.013, 0.015)	**<0.001**	0.002 (0.001, 0.003)	**0.001***	-0.0 (-0.001, 0.001)	0.43	0.0 (-0.001, 0.002)	0.91
	R	0.014 (0.013, 0.015)	**<0.001**	0.002 (0.001, 0.003)	**0.004***	-0.0 (-0.001, 0.001)	0.52	-0.0 (-0.001, 0.001)	0.99
Putamen	L	0.005 (0.005, 0.006)	**<0.001**	0.001 (0.0, 0.001)	**0.004***	-0.0 (-0.001, -0.0)	**0.03**	0.001 (-0.0, 0.001)	0.07
	R	0.006 (0.005, 0.006)	**<0.001**	0.001 (0.0, 0.002)	**0.003***	-0.0 (-0.001, 0.0)	0.13	0.001 (0.0, 0.002)	**0.006**
Thalamus	L	0.006 (0.005, 0.006)	**<0.001**	0.001 (0.0, 0.002)	**0.04***	-0.001 (-0.001, -0.0)	**0.005***	0.001 (-0.0, 0.002)	0.20
	R	0.006 (0.005, 0.006)	**<0.001**	0.001 (0.0, 0.002)	**0.01***	-0.001 (-0.001, -0.0)	**0.003***	0.0 (-0.001, 0.001)	0.36
Ventral DC	L	0.005 (0.005, 0.006)	**<0.001**	0.001 (0.0, 0.002)	**0.01***	-0.0 (-0.001, 0.0)	0.39	0.0 (-0.001, 0.001)	0.95
	R	0.005 (0.005, 0.006)	**<0.001**	0.001 (0.0, 0.002)	**0.03***	-0.001 (-0.001, 0.0)	0.07	-0.0 (-0.001, 0.001)	0.82

Note. Estimated regression coefficients (β), 95% confidence intervals (CI), and *p*-values from the linear mixed-effects models examining whether changes in BMI over time (i.e., age) led to changes in RNI. Models were controlled for the caregiver's highest education (effects coded) and framewise displacement. Random effects were modeled from subject ID and scanner serial number (e.g., MRI scanner ID). Significant effects are bolded, where * indicates that the association survived the Benjamini-Hochberg multiple comparison testing correction. BMI = body mass index. ROI=Region-of-interest; H=Hemisphere; L=Left; R=Right.

© 2025 Adise S et al. *JAMA Network Open*.

eTable 8. Results for Model 2 Among Males With Various Amounts of Weight Gain (ie, Relatively Weight Stable to Relatively Unhealthy Weight Gain)

BMI ~ RNI*Age + Puberty + Education + Motion + (1|MRI scanner ID) + (1|subject ID)

		Age		RNI		Age*RNI		Age	
**ROI**	**H**	**β (95% CI)**	***p***	**β (95% CI)**	***p***	**β (95% CI)**	***p***	**β (95% CI)**	***p***
Accumbens	L	0.73 (0.67, 0.79)	**<0.001**	0.12 (0.05, 0.2)	**0.001***	0.01 (-0.04, 0.07)	0.59	0.18 (0.09, 0.28)	**<0.001**
	R	0.73 (0.67, 0.79)	**<0.001**	0.11 (0.04, 0.19)	**0.003***	0.02 (-0.03, 0.07)	0.48	0.18 (0.08, 0.27)	**<0.001**
Amygdala	L	0.76 (0.7, 0.82)	**<0.001**	0.04 (-0.03, 0.11)	0.23	-0.01 (-0.06, 0.04)	0.58	0.2 (0.1, 0.29)	**<0.001**
	R	0.74 (0.68, 0.8)	**<0.001**	0.08 (0.01, 0.14)	**0.03**	0.01 (-0.04, 0.06)	0.63	0.19 (0.1, 0.28)	**<0.001**
Caudate	L	0.75 (0.69, 0.81)	**<0.001**	0.07 (-0.0, 0.13)	**0.05**	0.01 (-0.04, 0.06)	0.81	0.18 (0.09, 0.28)	**<0.001**
	R	0.75 (0.69, 0.81)	**<0.001**	0.08 (0.01, 0.15)	**0.02**	-0.03 (-0.08, 0.02)	0.19	0.19 (0.1, 0.28)	**<0.001**
Hippocampus	L	0.76 (0.71, 0.82)	**<0.001**	0.04 (-0.03, 0.11)	0.27	-0.02 (-0.07, 0.03)	0.44	0.2 (0.1, 0.29)	**<0.001**
	R	0.75 (0.7, 0.81)	**<0.001**	0.05 (-0.02, 0.12)	0.18	-0.03 (-0.08, 0.02)	0.25	0.2 (0.1, 0.29)	**<0.001**
Pallidum	L	0.72 (0.66, 0.78)	**<0.001**	0.11 (0.03, 0.18)	**0.006***	-0.0 (-0.05, 0.04)	0.89	0.2 (0.11, 0.29)	**<0.001**
	R	0.73 (0.66, 0.79)	**<0.001**	0.09 (0.01, 0.16)	**0.03**	-0.0 (-0.05, 0.05)	0.91	0.19 (0.1, 0.28)	**<0.001**
Putamen	L	0.74 (0.68, 0.81)	**<0.001**	0.06 (-0.02, 0.13)	0.14	-0.01 (-0.06, 0.04)	0.70	0.19 (0.1, 0.28)	**<0.001**
	R	0.73 (0.67, 0.79)	**<0.001**	0.09 (0.01, 0.16)	**0.02**	-0.0 (-0.05, 0.05)	0.91	0.16 (0.07, 0.26)	**0.001**
Thalamus	L	0.76 (0.7, 0.82)	**<0.001**	0.03 (-0.05, 0.1)	0.45	-0.04 (-0.09, 0.01)	0.14	0.2 (0.1, 0.29)	**<0.001**
	R	0.76 (0.7, 0.82)	**<0.001**	0.04 (-0.04, 0.11)	0.34	-0.04 (-0.09, 0.01)	0.11	0.2 (0.11, 0.29)	**<0.001**
Ventral DC	L	0.75 (0.69, 0.8)	**<0.001**	0.08 (0.0, 0.15)	**0.04**	0.01 (-0.04, 0.05)	0.82	0.19 (0.1, 0.29)	**<0.001**
	R	0.76 (0.7, 0.81)	**<0.001**	0.05 (-0.02, 0.13)	0.15	-0.0 (-0.06, 0.05)	0.87	0.19 (0.1, 0.29)	**<0.001**

Note. Estimated regression coefficients (β), 95% confidence intervals (CI), and *p*-values from the linear mixed-effects models examining whether changes in RNI over time (i.e., age) led to changes in BMI. Models were controlled for the caregiver's highest education (effects coded) and framewise displacement. Random effects were modeled from subject ID and scanner serial number (e.g., MRI scanner ID). Significant effects are bolded, where * indicates that the association survived the Benjamini-Hochberg multiple comparison testing correction. BMI = body mass index. ROI=Region-of-interest; H=Hemisphere; L=Left; R=Right.

© 2025 Adise S et al. *JAMA Network Open*.

eTable 9. Results for Model 1 for Females Who Were Classified as Healthy Weight, Weight Stable (HW-WS)

RNI ~ BMI*Age + Puberty + Education + Motion + (1|MRI scanner ID) + (1|subject ID)

		**Age**		**BMI**		**BMI*Age**		**Puberty**	
**ROI**	**H**	**β (95% CI)**	***p***	**β (95% CI)**	***p***	**β (95% CI)**	***p***	**β (95% CI)**	***p***
Accumbens	L	0.005 (0.004, 0.007)	**<0.001**	0.002 (-0.0, 0.004)	0.06	0.0 (-0.001, 0.001)	0.65	0.001 (-0.0, 0.002)	0.17
	R	0.006 (0.005, 0.008)	**<0.001**	0.002 (0.0, 0.004)	**0.02**	0.001 (-0.0, 0.002)	0.26	0.001 (-0.0, 0.002)	0.20
Amygdala	L	0.005 (0.004, 0.006)	**<0.001**	-0.0 (-0.002, 0.001)	0.85	0.001 (-0.0, 0.002)	0.16	0.001 (-0.0, 0.002)	0.17
	R	0.005 (0.004, 0.007)	**<0.001**	0.0 (-0.001, 0.002)	0.54	0.001 (0.0, 0.002)	**0.05**	0.001 (-0.001, 0.002)	0.32
Caudate	L	0.003 (0.002, 0.004)	**<0.001**	0.0 (-0.001, 0.002)	0.62	-0.001 (-0.001, 0.0)	0.16	0.001 (0.0, 0.002)	**0.04**
	R	0.004 (0.002, 0.005)	**<0.001**	0.001 (-0.0, 0.003)	0.07	0.0 (-0.001, 0.001)	0.67	0.001 (-0.0, 0.002)	0.20
Hippocampus	L	0.004 (0.003, 0.005)	**<0.001**	0.001 (-0.001, 0.002)	0.27	0.0 (-0.0, 0.001)	0.29	0.0 (-0.001, 0.001)	0.99
	R	0.005 (0.004, 0.005)	**<0.001**	0.002 (0.001, 0.003)	**0.006***	0.0 (-0.0, 0.001)	0.17	-0.0 (-0.001, 0.001)	0.73
Pallidum	L	0.014 (0.013, 0.016)	**<0.001**	0.002 (-0.001, 0.005)	0.19	-0.0 (-0.001, 0.001)	0.95	0.002 (-0.0, 0.004)	0.06
	R	0.015 (0.013, 0.017)	**<0.001**	0.001 (-0.002, 0.003)	0.69	-0.0 (-0.001, 0.001)	0.90	0.0 (-0.001, 0.002)	0.59
Putamen	L	0.005 (0.005, 0.006)	**<0.001**	0.002 (0.001, 0.003)	**0.006***	0.0 (-0.0, 0.001)	0.53	0.001 (-0.0, 0.002)	0.11
	R	0.006 (0.005, 0.007)	**<0.001**	0.002 (0.0, 0.003)	**0.01***	0.0 (-0.0, 0.001)	0.41	0.001 (-0.0, 0.002)	0.15
Thalamus	L	0.006 (0.005, 0.007)	**<0.001**	0.001 (-0.001, 0.003)	0.41	0.0 (-0.001, 0.001)	0.82	0.0 (-0.001, 0.001)	0.67
	R	0.007 (0.006, 0.008)	**<0.001**	0.002 (0.0, 0.004)	**0.03**	0.001 (-0.0, 0.002)	0.09	-0.0 (-0.001, 0.001)	0.85
Ventral DC	L	0.005 (0.004, 0.006)	**<0.001**	0.001 (-0.0, 0.003)	0.07	0.001 (-0.0, 0.002)	0.26	0.0 (-0.001, 0.001)	0.97
	R	0.005 (0.004, 0.007)	**<0.001**	0.002 (0.0, 0.004)	**0.01***	0.001 (-0.0, 0.002)	0.25	-0.0 (-0.002, 0.001)	0.64

Note. Estimated regression coefficients (β), 95% confidence intervals (CI), and *p*-values from the linear mixed-effects models examining whether changes in BMI over time (i.e., age) led to changes in RNI. Models were controlled for the caregiver's highest education (effects coded) and framewise displacement. Random effects were modeled from subject ID and scanner serial number (e.g., MRI scanner ID). Significant effects are bolded, where * indicates that the association survived the Benjamini-Hochberg multiple comparison testing correction. BMI = body mass index. ROI=Region-of-interest; H=Hemisphere; L=Left; R=Right.

© 2025 Adise S et al. *JAMA Network Open*.

eTable 10. Results for Model 2 for Females Who Were Classified as Healthy Weight, Weight Stable (HW-WS)

BMI ~ RNI*Age + Puberty + Education + Motion + (1|MRI scanner ID)

		Age		RNI		RNI*Age		Puberty	
**ROI**	**H**	**β (95% CI)**	***p***	**β (95% CI)**	***p***	**β (95% CI)**	***p***	**β (95% CI)**	***p***
Accumbens	L	0.37 (0.31, 0.43)	**<0.001**	0.03 (-0.04, 0.09)	0.45	0.02 (-0.01, 0.06)	0.23	0.18 (0.12, 0.25)	**<0.001**
	R	0.36 (0.3, 0.42)	**<0.001**	0.06 (-0.01, 0.12)	0.08	0.02 (-0.02, 0.05)	0.29	0.18 (0.11, 0.24)	**<0.001**
Amygdala	L	0.36 (0.3, 0.42)	**<0.001**	0.04 (-0.02, 0.1)	0.16	0.03 (-0.01, 0.06)	0.14	0.17 (0.1, 0.23)	**<0.001**
	R	0.36 (0.3, 0.42)	**<0.001**	0.04 (-0.02, 0.1)	0.22	0.05 (0.01, 0.08)	**0.009**	0.18 (0.11, 0.24)	**<0.001**
Caudate	L	0.37 (0.31, 0.43)	**<0.001**	0.01 (-0.05, 0.07)	0.74	-0.03 (-0.06, 0.01)	0.16	0.18 (0.12, 0.25)	**<0.001**
	R	0.37 (0.31, 0.43)	**<0.001**	0.03 (-0.03, 0.09)	0.29	-0.0 (-0.04, 0.03)	0.82	0.17 (0.11, 0.24)	**<0.001**
Hippocampus	L	0.37 (0.31, 0.43)	**<0.001**	0.03 (-0.03, 0.1)	0.36	0.01 (-0.02, 0.05)	0.50	0.17 (0.1, 0.24)	**<0.001**
	R	0.35 (0.28, 0.41)	**<0.001**	0.09 (0.02, 0.16)	**0.01**	0.02 (-0.02, 0.05)	0.30	0.18 (0.11, 0.24)	**<0.001**
Pallidum	L	0.37 (0.3, 0.44)	**<0.001**	0.01 (-0.08, 0.09)	0.88	0.01 (-0.03, 0.04)	0.66	0.18 (0.11, 0.24)	**<0.001**
	R	0.37 (0.31, 0.44)	**<0.001**	-0.01 (-0.09, 0.08)	0.89	-0.02 (-0.05, 0.02)	0.41	0.18 (0.12, 0.25)	**<0.001**
Putamen	L	0.35 (0.29, 0.41)	**<0.001**	0.06 (-0.02, 0.13)	0.13	0.01 (-0.02, 0.05)	0.44	0.18 (0.11, 0.24)	**<0.001**
	R	0.35 (0.28, 0.41)	**<0.001**	0.06 (-0.01, 0.13)	0.09	0.02 (-0.02, 0.06)	0.26	0.17 (0.11, 0.24)	**<0.001**
Thalamus	L	0.37 (0.31, 0.43)	**<0.001**	0.03 (-0.05, 0.1)	0.49	-0.01 (-0.04, 0.03)	0.63	0.18 (0.11, 0.24)	**<0.001**
	R	0.36 (0.29, 0.42)	**<0.001**	0.06 (-0.01, 0.13)	0.12	-0.01 (-0.04, 0.03)	0.69	0.18 (0.12, 0.25)	**<0.001**
Ventral DC	L	0.36 (0.3, 0.42)	**<0.001**	0.05 (-0.02, 0.12)	0.19	-0.0 (-0.04, 0.04)	0.99	0.18 (0.11, 0.24)	**<0.001**
	R	0.36 (0.29, 0.42)	**<0.001**	0.06 (-0.01, 0.12)	0.12	0.01 (-0.03, 0.04)	0.66	0.18 (0.11, 0.24)	**<0.001**

Note. Estimated regression coefficients (β), 95% confidence intervals (CI), and *p*-values from the linear mixed-effects models examining whether changes in RNI over time (i.e., age) led to changes in BMI. Models were controlled for the caregiver highest education (effects coded) and framewise displacement. A random effect was modeled from subject ID but scanner was not included due to issues with Singularity. Significant effects are bolded, where * indicates that the association survived the Benjamini-Hochberg multiple comparison testing correction. BMI = body mass index. ROI=Region-of-interest; H=Hemisphere; L=Left; R=Right.

© 2025 Adise S et al. *JAMA Network Open*.

eTable 11. Results for Model 1 for Females With Various Amounts of Weight Gain (ie, Relatively Weight Stable to Relatively Unhealthy Weight Gain)

RNI ~ BMI*Age + Puberty + Education + Motion + (1|MRI scanner ID) + (1|subject ID)

		**Age**		**BMI**		**BMI*Age**		**Puberty**	
**ROI**	**H**	**β (95% CI)**	***p***	**β (95% CI)**	***p***	**β (95% CI)**	***p***	**β (95% CI)**	***p***
Accumbens	L	0.005 (0.004, 0.006)	**<0.001**	0.002 (0.002, 0.003)	**<0.001***	-0.0 (-0.001, 0.0)	0.37	0.001 (-0.0, 0.001)	0.20
	R	0.006 (0.005, 0.007)	**<0.001**	0.002 (0.001, 0.003)	**<0.001***	-0.0 (-0.001, 0.0)	0.18	0.001 (-0.0, 0.001)	0.13
Amygdala	L	0.005 (0.004, 0.006)	**<0.001**	-0.0 (-0.001, 0.001)	0.63	0.0 (-0.0, 0.001)	0.47	0.0 (-0.0, 0.001)	0.40
	R	0.005 (0.005, 0.006)	**<0.001**	0.0 (-0.001, 0.001)	0.66	0.0 (-0.0, 0.001)	0.71	0.0 (-0.001, 0.001)	0.78
Caudate	L	0.003 (0.003, 0.004)	**<0.001**	0.001 (0.001, 0.002)	**<0.001***	-0.001 (-0.001, -0.0)	**0.02**	0.0 (-0.0, 0.001)	0.45
	R	0.004 (0.003, 0.005)	**<0.001**	0.002 (0.001, 0.003)	**<0.001***	-0.001 (-0.001, -0.0)	**0.01**	-0.0 (-0.001, 0.001)	0.78
Hippocampus	L	0.004 (0.004, 0.005)	**<0.001**	0.001 (0.0, 0.002)	**0.001***	-0.0 (-0.001, 0.0)	0.44	-0.0 (-0.001, 0.0)	0.42
	R	0.005 (0.004, 0.005)	**<0.001**	0.001 (0.0, 0.002)	**0.001***	0.0 (-0.0, 0.0)	0.71	0.0 (-0.001, 0.001)	0.89
Pallidum	L	0.014 (0.013, 0.015)	**<0.001**	0.002 (0.001, 0.004)	**<0.001***	-0.0 (-0.001, 0.0)	0.28	0.0 (-0.001, 0.001)	0.55
	R	0.014 (0.013, 0.015)	**<0.001**	0.002 (0.001, 0.003)	**0.002***	-0.001 (-0.002, -0.0)	**0.02**	-0.0 (-0.001, 0.001)	0.77
Putamen	L	0.006 (0.005, 0.007)	**<0.001**	0.001 (0.001, 0.002)	**<0.001***	-0.0 (-0.001, 0.0)	**0.05**	0.0 (-0.0, 0.001)	0.63
	R	0.006 (0.006, 0.007)	**<0.001**	0.001 (0.001, 0.002)	**<0.001***	-0.0 (-0.001, 0.0)	0.07	0.0 (-0.0, 0.001)	0.53
Thalamus	L	0.006 (0.005, 0.006)	**<0.001**	0.001 (0.001, 0.002)	**<0.001***	-0.0 (-0.001, 0.0)	0.25	-0.0 (-0.001, 0.001)	0.83
	R	0.006 (0.006, 0.007)	**<0.001**	0.002 (0.001, 0.003)	**<0.001***	-0.0 (-0.001, 0.0)	0.82	-0.0 (-0.001, 0.001)	0.72
Ventral DC	L	0.005 (0.004, 0.006)	**<0.001**	0.001 (0.0, 0.002)	**0.02***	0.0 (-0.0, 0.001)	0.82	0.0 (-0.001, 0.001)	0.52
	R	0.005 (0.004, 0.006)	**<0.001**	0.001 (0.0, 0.002)	**0.001***	-0.0 (-0.001, 0.0)	0.82	-0.0 (-0.001, 0.0)	0.47

Note. Estimated regression coefficients (β), 95% confidence intervals (CI), and *p*-values from the linear mixed-effects models examining whether changes in BMI over time (i.e., age) led to changes in RNI. Models were controlled for the caregiver's highest education (effects coded) and framewise displacement. Random effects were modeled from subject ID and scanner serial number (e.g., MRI scanner ID). Significant effects are bolded, where * indicates that the association survived the Benjamini-Hochberg multiple comparison testing correction. BMI = body mass index. ROI=Region-of-interest; H=Hemisphere; L=Left; R=Right.

© 2025 Adise S et al. *JAMA Network Open*.

eTable 12. Results for Model 2 for Females With Various Amounts of Weight Gain (ie, Relatively Weight Stable to Relatively Unhealthy Weight Gain)

BMI ~ RNI*Age + Puberty + Education + Motion + (1|MRI scanner ID) + (1|subject ID)

		Age		RNI		RNI*Age		Puberty	
**ROI**	**H**	**β (95% CI)**	***p***	**β (95% CI)**	***p***	**β (95% CI)**	***p***	**β (95% CI)**	***p***
Accumbens	L	0.72 (0.64, 0.8)	**<0.001**	0.18 (0.11, 0.26)	**<0.001***	0.13 (0.08, 0.18)	**<0.001***	0.4 (0.32, 0.47)	**<0.001**
	R	0.72 (0.64, 0.8)	**<0.001**	0.14 (0.07, 0.21)	**<0.001***	0.13 (0.08, 0.18)	**<0.001***	0.4 (0.32, 0.48)	**<0.001**
Amygdala	L	0.78 (0.7, 0.86)	**<0.001**	-0.0 (-0.07, 0.07)	0.94	0.07 (0.02, 0.12)	**0.007***	0.4 (0.32, 0.48)	**<0.001**
	R	0.77 (0.69, 0.85)	**<0.001**	0.01 (-0.06, 0.08)	0.87	0.08 (0.03, 0.13)	**0.002***	0.4 (0.32, 0.47)	**<0.001**
Caudate	L	0.74 (0.67, 0.82)	**<0.001**	0.11 (0.04, 0.18)	**0.002***	0.05 (-0.0, 0.1)	0.06	0.4 (0.32, 0.48)	**<0.001**
	R	0.74 (0.66, 0.81)	**<0.001**	0.13 (0.06, 0.2)	**<0.001***	0.09 (0.04, 0.14)	**0.001***	0.39 (0.32, 0.47)	**<0.001**
Hippocampus	L	0.74 (0.66, 0.82)	**<0.001**	0.12 (0.05, 0.2)	**0.001***	0.08 (0.03, 0.13)	**0.002***	0.39 (0.32, 0.47)	**<0.001**
	R	0.73 (0.65, 0.81)	**<0.001**	0.12 (0.05, 0.2)	**0.001***	0.07 (0.02, 0.12)	**0.008***	0.39 (0.32, 0.47)	**<0.001**
Pallidum	L	0.72 (0.64, 0.8)	**<0.001**	0.12 (0.04, 0.21)	**0.003***	0.11 (0.06, 0.16)	**<0.001***	0.39 (0.31, 0.47)	**<0.001**
	R	0.74 (0.66, 0.82)	**<0.001**	0.06 (-0.02, 0.14)	0.13	0.06 (0.01, 0.12)	**0.017***	0.4 (0.32, 0.48)	**<0.001**
Putamen	L	0.7 (0.61, 0.78)	**<0.001**	0.15 (0.07, 0.22)	**<0.001***	0.14 (0.09, 0.19)	**<0.001***	0.4 (0.32, 0.48)	**<0.001**
	R	0.7 (0.62, 0.78)	**<0.001**	0.11 (0.04, 0.19)	**0.004***	0.12 (0.07, 0.17)	**<0.001***	0.4 (0.32, 0.48)	**<0.001**
Thalamus	L	0.76 (0.68, 0.84)	**<0.001**	0.1 (0.02, 0.18)	**0.01***	0.02 (-0.03, 0.08)	0.35	0.4 (0.32, 0.48)	**<0.001**
	R	0.73 (0.65, 0.81)	**<0.001**	0.13 (0.06, 0.21)	**0.001***	0.07 (0.02, 0.12)	**0.01***	0.4 (0.32, 0.48)	**<0.001**
Ventral DC	L	0.76 (0.68, 0.84)	**<0.001**	0.05 (-0.02, 0.13)	0.18	0.04 (-0.01, 0.1)	0.11	0.41 (0.33, 0.48)	**<0.001**
	R	0.76 (0.68, 0.84)	**<0.001**	0.07 (-0.0, 0.15)	0.06	0.04 (-0.02, 0.09)	0.17	0.4 (0.32, 0.48)	**<0.001**

Note. Estimated regression coefficients (β), 95% confidence intervals (CI), and *p*-values from the linear mixed-effects models examining whether changes in RNI over time (i.e., age) led to changes in BMI. Models were controlled for the caregiver's highest education (effects coded) and framewise displacement. Random effects were modeled from subject ID and scanner serial number (e.g., MRI scanner ID). Significant effects are bolded, where * indicates that the association survived the Benjamini-Hochberg multiple comparison testing correction. BMI = body mass index. ROI=Region-of-interest; H=Hemisphere; L=Left; R=Right.

eTable 13. Results From the Logistic Regression for Model 1 in Males at Baseline

RNI ~ Age + Group (HW-WS vs. HW-NS) + Puberty + Education + Motion + (1|MRI scanner ID)

		Age		Group		Puberty	
**ROI**	**H**	**β (95% CI)**	***p***	**β (95% CI)**	***p***	**β (95% CI)**	***p***
Accumbens	L	0.007 (0.005, 0.009)	**<0.001**	0.001 (-0.001, 0.003)	0.34	0.001 (-0.001, 0.003)	0.34
	R	0.007 (0.005, 0.009)	**<0.001**	0.003 (0.001, 0.005)	**0.01**	0.001 (-0.001, 0.003)	0.32
Amygdala	L	0.005 (0.004, 0.007)	**<0.001**	-0.0 (-0.002, 0.002)	0.71	-0.0 (-0.002, 0.001)	0.69
	R	0.006 (0.004, 0.007)	**<0.001**	0.001 (-0.001, 0.003)	0.33	0.0 (-0.002, 0.002)	0.96
Caudate	L	0.006 (0.004, 0.007)	**<0.001**	0.0 (-0.002, 0.002)	0.74	0.001 (-0.001, 0.003)	0.23
	R	0.005 (0.003, 0.007)	**<0.001**	0.001 (-0.001, 0.003)	0.32	0.0 (-0.002, 0.002)	0.85
Hippocampus	L	0.005 (0.003, 0.006)	**<0.001**	-0.0 (-0.002, 0.002)	0.86	0.0 (-0.001, 0.002)	0.84
	R	0.006 (0.004, 0.007)	**<0.001**	0.0 (-0.002, 0.002)	0.98	-0.0 (-0.002, 0.001)	0.73
Pallidum	L	0.017 (0.014, 0.02)	**<0.001**	0.003 (-0.001, 0.006)	0.15	-0.001 (-0.004, 0.002)	0.53
	R	0.017 (0.014, 0.019)	**<0.001**	0.002 (-0.001, 0.005)	0.17	-0.001 (-0.003, 0.002)	0.62
Putamen	L	0.007 (0.005, 0.008)	**<0.001**	0.001 (-0.001, 0.002)	0.32	0.001 (-0.001, 0.002)	0.27
	R	0.007 (0.005, 0.008)	**<0.001**	0.001 (-0.001, 0.002)	0.32	0.001 (-0.0, 0.002)	0.15
Thalamus	L	0.008 (0.007, 0.01)	**<0.001**	0.0 (-0.002, 0.003)	0.72	0.002 (-0.0, 0.004)	0.07
	R	0.008 (0.006, 0.009)	**<0.001**	0.001 (-0.001, 0.003)	0.27	0.001 (-0.0, 0.003)	0.14
Ventral DC	L	0.007 (0.006, 0.009)	**<0.001**	0.0 (-0.002, 0.002)	0.79	-0.0 (-0.002, 0.002)	1.00
	R	0.007 (0.006, 0.009)	**<0.001**	0.001 (-0.001, 0.003)	0.21	-0.0 (-0.002, 0.002)	0.92

Note. Estimated regression coefficients (β), 95% confidence intervals (CI), and *p*-values from the logistic mixed effects models examining whether group (e.g., healthy weight, weight stable [HWWS], weight gain [WG] predicted RNI at baseline (ages 9/10-years-old). Models were controlled for the caregiver's highest education (effects coded) and framewise displacement. A random intercept was modeled for scanner serial number (e.g., MRI scanner ID). Given that group was time invariant, models were conducted separately for each time point. Significant effects are bolded, where * indicates that the association survived the Benjamini-Hochberg multiple comparison testing correction. BMI = body mass index. ROI=Region-of-interest; H=Hemisphere; L=Left; R=Right.

© 2025 Adise S et al. *JAMA Network Open*.

eTable 14. Results From the Logistic Regression for Model 1 in Males at Year 2

RNI ~ Age + Group (HW-WS vs. HW-NS) + Puberty + Education + Motion + (1|MRI scanner ID)

		Age		Group		Puberty	
**ROI**	**H**	**β (95% CI)**	***p***	**β (95% CI)**	***p***	**β (95% CI)**	***p***
Accumbens	L	0.006 (0.004, 0.008)	**<0.001**	0.001 (-0.001, 0.003)	0.42	0.003 (0.001, 0.004)	**0.001**
	R	0.006 (0.004, 0.008)	**<0.001**	0.002 (-0.0, 0.004)	0.09	0.002 (0.001, 0.004)	**0.008**
Amygdala	L	0.005 (0.003, 0.007)	**<0.001**	-0.001 (-0.003, 0.001)	0.50	-0.0 (-0.002, 0.001)	0.88
	R	0.005 (0.003, 0.007)	**<0.001**	-0.0 (-0.002, 0.002)	0.75	-0.0 (-0.002, 0.001)	0.67
Caudate	L	0.005 (0.003, 0.007)	**<0.001**	0.001 (-0.001, 0.003)	0.22	0.001 (-0.001, 0.002)	0.24
	R	0.004 (0.002, 0.006)	**<0.001**	0.0 (-0.002, 0.002)	0.80	0.001 (-0.0, 0.002)	0.21
Hippocampus	L	0.004 (0.003, 0.006)	**<0.001**	-0.0 (-0.002, 0.002)	0.97	0.0 (-0.001, 0.001)	0.95
	R	0.004 (0.002, 0.005)	**<0.001**	-0.0 (-0.002, 0.001)	0.83	-0.0 (-0.002, 0.001)	0.56
Pallidum	L	0.015 (0.012, 0.018)	**<0.001**	0.002 (-0.001, 0.006)	0.25	0.003 (0.0, 0.005)	**0.03**
	R	0.014 (0.01, 0.017)	**<0.001**	0.003 (-0.001, 0.006)	0.12	0.002 (-0.001, 0.004)	0.14
Putamen	L	0.006 (0.004, 0.007)	**<0.001**	-0.0 (-0.002, 0.001)	0.84	0.001 (0.0, 0.003)	**0.02**
	R	0.005 (0.004, 0.007)	**<0.001**	0.0 (-0.001, 0.002)	0.66	0.002 (0.001, 0.003)	**0.001**
Thalamus	L	0.006 (0.004, 0.008)	**<0.001**	0.001 (-0.001, 0.003)	0.50	0.001 (-0.0, 0.003)	0.14
	R	0.006 (0.004, 0.008)	**<0.001**	-0.0 (-0.002, 0.002)	0.72	0.001 (-0.0, 0.003)	0.15
Ventral DC	L	0.006 (0.004, 0.008)	**<0.001**	0.0 (-0.002, 0.002)	0.92	0.001 (-0.001, 0.002)	0.25
	R	0.005 (0.003, 0.006)	**<0.001**	-0.0 (-0.002, 0.002)	0.74	0.0 (-0.001, 0.002)	0.64

Note. Estimated regression coefficients (β), 95% confidence intervals (CI), and *p*-values from the logistic mixed effects models examining whether group (e.g., healthy weight, weight stable [HWWS], weight gain [WG]) predicted RNI at year 2 (ages 11/12-years-old). Models were controlled for the caregiver's highest education (effects coded) and framewise displacement. A random intercept was modeled for scanner serial number (e.g., MRI scanner ID). Given that group was time invariant, models were conducted separately for each time point. Significant effects are bolded, where * indicates that the association survived the Benjamini-Hochberg multiple comparison testing correction. BMI = body mass index. ROI=Region-of-interest; H=Hemisphere; L=Left; R=Right.

© 2025 Adise S et al. *JAMA Network Open*.

eTable 15. Results From the Logistic Regression for Model 2 in Males at Baseline

Group (HW-WS vs. HW-NS) ~ RNI + Age + Puberty + Education + Motion + (1|MRI scanner ID)

		Age		RNI		Puberty	
**ROI**	**H**	**β (95% CI)**	***p***	**β (95% CI)**	***p***	**β (95% CI)**	***p***
Accumbens	L	-0.004 (-0.271, 0.262)	0.97	0.078 (-0.093, 0.248)	0.37	-0.151 (-0.4, 0.098)	0.23
	R	-0.023 (-0.292, 0.246)	0.87	0.185 (0.014, 0.356)	**0.03**	-0.157 (-0.407, 0.093)	0.22
Amygdala	L	0.044 (-0.22, 0.308)	0.75	0.0 (-0.151, 0.151)	1.00	-0.157 (-0.405, 0.092)	0.22
	R	0.025 (-0.24, 0.291)	0.85	0.083 (-0.075, 0.24)	0.30	-0.169 (-0.417, 0.08)	0.18
Caudate	L	0.028 (-0.236, 0.292)	0.84	0.064 (-0.087, 0.214)	0.41	-0.168 (-0.416, 0.081)	0.19
	R	0.011 (-0.254, 0.276)	0.93	0.063 (-0.087, 0.213)	0.41	-0.182 (-0.434, 0.071)	0.16
Hippocampus	L	0.032 (-0.233, 0.296)	0.81	0.019 (-0.128, 0.166)	0.80	-0.153 (-0.4, 0.095)	0.23
	R	0.031 (-0.235, 0.296)	0.82	0.024 (-0.126, 0.173)	0.76	-0.17 (-0.417, 0.078)	0.18
Pallidum	L	-0.017 (-0.291, 0.257)	0.91	0.063 (-0.108, 0.234)	0.47	-0.159 (-0.409, 0.091)	0.21
	R	-0.021 (-0.296, 0.253)	0.88	0.101 (-0.076, 0.277)	0.27	-0.146 (-0.395, 0.102)	0.25
Putamen	L	-0.024 (-0.294, 0.246)	0.86	0.094 (-0.068, 0.256)	0.25	-0.156 (-0.405, 0.094)	0.22
	R	-0.021 (-0.292, 0.25)	0.88	0.109 (-0.057, 0.276)	0.20	-0.162 (-0.412, 0.087)	0.20
Thalamus	L	-0.013 (-0.282, 0.256)	0.93	0.036 (-0.122, 0.195)	0.65	-0.149 (-0.398, 0.101)	0.24
	R	-0.023 (-0.291, 0.245)	0.87	0.043 (-0.12, 0.205)	0.61	-0.161 (-0.412, 0.09)	0.21
Ventral DC	L	0.013 (-0.254, 0.28)	0.92	0.016 (-0.144, 0.176)	0.85	-0.162 (-0.411, 0.086)	0.20
	R	-0.0 (-0.268, 0.267)	1.00	0.084 (-0.086, 0.253)	0.33	-0.145 (-0.393, 0.103)	0.25

Note. Estimated regression coefficients (β), 95% confidence intervals (CI), and *p*-values from the logistic mixed effects models examining whether RNI at baseline predicted group membership (e.g., healthy weight, weight stable [HW-WS], [initially] healthy weight, not-stable [HW-NS]). Models were controlled for the caregiver's highest education (effects coded) and framewise displacement. A random intercept was modeled for scanner serial number (e.g., MRI scanner ID). Given that group was time invariant, models were conducted separately for each time point. Significant effects are bolded, where * indicates that the association survived the Benjamini-Hochberg multiple comparison testing correction. BMI = body mass index. ROI=Region-of-interest; H=Hemisphere; L=Left; R=Right.

© 2025 Adise S et al. *JAMA Network Open*.

eTable 16. Results From the Logistic Regression for Model 2 in Males at Year 2

Group (HW-WS vs. HW-NS) ~ RNI + Age + Education + Motion + (1|MRI scanner ID)

		Age		RNI		Puberty	
**ROI**	**H**	**β (95% CI)**	***p***	**β (95% CI)**	***p***	**β (95% CI)**	***p***
Accumbens	L	-0.031 (-0.303, 0.241)	0.82	-0.015 (-0.179, 0.148)	0.85	0.133 (-0.071, 0.337)	0.20
	R	-0.043 (-0.317, 0.231)	0.76	0.053 (-0.112, 0.218)	0.53	0.123 (-0.082, 0.327)	0.24
Amygdala	L	0.022 (-0.251, 0.295)	0.87	-0.083 (-0.239, 0.074)	0.30	0.116 (-0.087, 0.319)	0.26
	R	-0.021 (-0.296, 0.253)	0.88	-0.05 (-0.203, 0.103)	0.52	0.139 (-0.064, 0.342)	0.18
Caudate	L	-0.022 (-0.293, 0.25)	0.88	0.075 (-0.074, 0.224)	0.32	0.127 (-0.074, 0.329)	0.22
	R	-0.005 (-0.276, 0.266)	0.97	0.006 (-0.151, 0.162)	0.94	0.127 (-0.075, 0.329)	0.22
Hippocampus	L	0.006 (-0.267, 0.28)	0.96	-0.006 (-0.168, 0.156)	0.94	0.126 (-0.076, 0.328)	0.22
	R	-0.003 (-0.277, 0.271)	0.98	-0.039 (-0.198, 0.119)	0.63	0.128 (-0.076, 0.332)	0.22
Pallidum	L	-0.059 (-0.34, 0.222)	0.68	0.042 (-0.132, 0.216)	0.64	0.151 (-0.053, 0.356)	0.15
	R	-0.046 (-0.325, 0.234)	0.75	0.066 (-0.104, 0.236)	0.45	0.141 (-0.063, 0.344)	0.18
Putamen	L	0.009 (-0.265, 0.283)	0.95	-0.051 (-0.217, 0.115)	0.55	0.144 (-0.059, 0.347)	0.16
	R	-0.004 (-0.278, 0.27)	0.98	-0.017 (-0.185, 0.151)	0.84	0.121 (-0.085, 0.328)	0.25
Thalamus	L	-0.032 (-0.307, 0.243)	0.82	0.006 (-0.16, 0.172)	0.94	0.152 (-0.051, 0.355)	0.14
	R	-0.009 (-0.283, 0.265)	0.95	-0.064 (-0.239, 0.11)	0.47	0.14 (-0.064, 0.343)	0.18
Ventral DC	L	-0.011 (-0.285, 0.262)	0.93	-0.033 (-0.196, 0.13)	0.69	0.149 (-0.053, 0.352)	0.15
	R	-0.005 (-0.277, 0.267)	0.97	-0.057 (-0.223, 0.108)	0.50	0.149 (-0.054, 0.352)	0.15

Note. Estimated regression coefficients (β), 95% confidence intervals (CI), and *p*-values from the logistic mixed effects models examining the association whether RNI at baseline predicted group membership (e.g., healthy weight, weight stable [HW-WS], [initially] healthy weight, not-stable [HW-NS]). Models were controlled for the caregiver's highest education (effects coded) and framewise displacement. A random intercept was modeled for scanner serial number (e.g., MRI scanner ID). Given that group was time invariant, models were conducted separately for each time point. Significant effects are bolded, where * indicates that the association survived the Benjamini-Hochberg multiple comparison testing correction. BMI = body mass index. ROI=Region-of-interest; H=Hemisphere; L=Left; R=Right.

© 2025 Adise S et al. *JAMA Network Open*.

eTable 17. Results From the Logistic Regression for Model 1 in Females at Baseline

RNI ~ Group (HW-WS vs. HW-NS) + Age + Puberty + Education + Motion + (1|MRI scanner ID)

		Age		Group		Puberty	
**ROI**	**H**	**β (95% CI)**	***p***	**β (95% CI)**	***p***	**β (95% CI)**	***p***
Accumbens	L	0.006 (0.004, 0.009)	**<0.001**	0.004 (0.001, 0.006)	**0.001***	0.001 (-0.0, 0.002)	0.11
	R	0.006 (0.004, 0.009)	**<0.001**	0.004 (0.002, 0.006)	**<0.001***	0.001 (-0.0, 0.002)	0.15
Amygdala	L	0.004 (0.002, 0.006)	**<0.001**	0.001 (-0.001, 0.003)	0.51	0.0 (-0.001, 0.002)	0.42
	R	0.004 (0.003, 0.006)	**<0.001**	0.002 (-0.0, 0.003)	0.09	0.0 (-0.001, 0.001)	0.83
Caudate	L	0.003 (0.002, 0.005)	**<0.001**	0.003 (0.001, 0.005)	**0.005***	0.0 (-0.001, 0.001)	0.81
	R	0.006 (0.004, 0.008)	**<0.001**	0.002 (0.001, 0.004)	**0.01***	-0.0 (-0.001, 0.001)	0.71
Hippocampus	L	0.004 (0.002, 0.006)	**<0.001**	0.002 (0.0, 0.004)	**0.01***	-0.001 (-0.001, 0.0)	0.26
	R	0.004 (0.003, 0.006)	**<0.001**	0.001 (-0.0, 0.003)	0.09	-0.0 (-0.001, 0.001)	0.53
Pallidum	L	0.014 (0.011, 0.017)	**<0.001**	0.004 (0.001, 0.007)	**0.007***	0.0 (-0.002, 0.002)	0.77
	R	0.015 (0.012, 0.018)	**<0.001**	0.005 (0.002, 0.008)	**0.003***	-0.0 (-0.002, 0.001)	0.66
Putamen	L	0.007 (0.005, 0.008)	**<0.001**	0.002 (0.001, 0.004)	**0.008***	0.0 (-0.0, 0.001)	0.38
	R	0.007 (0.006, 0.009)	**<0.001**	0.002 (0.001, 0.004)	**0.007***	0.001 (-0.0, 0.001)	0.25
Thalamus	L	0.006 (0.003, 0.008)	**<0.001**	-0.0 (-0.002, 0.002)	0.81	0.001 (-0.001, 0.002)	0.38
	R	0.006 (0.004, 0.008)	**<0.001**	0.001 (-0.001, 0.003)	0.39	0.0 (-0.001, 0.001)	0.59
Ventral DC	L	0.005 (0.003, 0.007)	**<0.001**	0.001 (-0.001, 0.002)	0.58	0.0 (-0.001, 0.002)	0.44
	R	0.005 (0.003, 0.007)	**<0.001**	0.001 (-0.001, 0.003)	0.60	-0.0 (-0.001, 0.001)	0.96

Note. Estimated regression coefficients (β), 95% confidence intervals (CI), and *p*-values from the logistic mixed effects models examining whether group (e.g., healthy weight, weight stable [HWWS], weight gain [WG] predicted RNI at baseline (ages 9/10-years-old). Models were controlled for the caregiver's highest education (effects coded) and framewise displacement. A random intercept was modeled for scanner serial number (e.g., MRI scanner ID). Given that group was time invariant, models were conducted separately for each time point. Significant effects are bolded, where * indicates that the association survived the Benjamini-Hochberg multiple comparison testing correction. BMI = body mass index. ROI=Region-of-interest; H=Hemisphere; L=Left; R=Right.

© 2025 Adise S et al. *JAMA Network Open*.

eTable 18. Results From the Logistic Regression for Model 1 in Females at Year 2

RNI ~ Group (HW-WS vs. HW-NS) + Age + Puberty + Education + Motion + (1|MRI scanner ID)

		**Age**		**Group**		**Puberty**	
**ROI**	**H**	**β (95% CI)**	***p***	**β (95% CI)**	***p***	**β (95% CI)**	***p***
Accumbens	L	0.005 (0.002, 0.007)	**<0.001**	0.003 (0.001, 0.005)	**0.02**	0.003 (0.001, 0.004)	**<0.001**
	R	0.005 (0.002, 0.007)	**<0.001**	0.003 (0.001, 0.006)	**0.01**	0.004 (0.002, 0.005)	**<0.001**
Amygdala	L	0.004 (0.002, 0.006)	**<0.001**	0.0 (-0.002, 0.002)	0.70	0.001 (-0.0, 0.003)	0.07
	R	0.003 (0.001, 0.005)	**0.001**	0.001 (-0.001, 0.003)	0.28	0.001 (-0.0, 0.002)	0.12
Caudate	L	0.003 (0.001, 0.005)	**0.001**	0.002 (0.0, 0.004)	**0.04**	0.001 (-0.0, 0.003)	**0.05**
	R	0.003 (0.001, 0.005)	**0.002**	0.002 (0.001, 0.004)	**0.01**	0.002 (0.0, 0.003)	**0.02**
Hippocampus	L	0.003 (0.002, 0.005)	**<0.001**	0.002 (0.001, 0.004)	**0.003***	0.001 (-0.0, 0.002)	0.13
	R	0.004 (0.002, 0.005)	**<0.001**	0.002 (0.0, 0.004)	**0.02**	0.001 (0.0, 0.002)	**0.02**
Pallidum	L	0.012 (0.009, 0.016)	**<0.001**	0.003 (-0.0, 0.007)	0.08	0.003 (0.0, 0.005)	**0.03**
	R	0.01 (0.006, 0.013)	**<0.001**	0.003 (-0.0, 0.007)	0.08	0.002 (0.0, 0.005)	**0.05**
Putamen	L	0.006 (0.004, 0.008)	**<0.001**	0.002 (0.0, 0.004)	**0.01**	0.001 (0.0, 0.002)	**0.03**
	R	0.005 (0.004, 0.007)	**<0.001**	0.002 (0.0, 0.003)	**0.03**	0.001 (0.0, 0.002)	**0.009**
Thalamus	L	0.005 (0.003, 0.007)	**<0.001**	0.0 (-0.002, 0.003)	0.69	0.001 (-0.0, 0.003)	0.08
	R	0.004 (0.002, 0.006)	**<0.001**	0.001 (-0.001, 0.003)	0.32	0.002 (0.0, 0.003)	**0.01**
Ventral DC	L	0.005 (0.003, 0.007)	**<0.001**	0.001 (-0.001, 0.003)	0.41	0.001 (0.0, 0.003)	**0.04**
	R	0.003 (0.001, 0.006)	**0.001**	0.001 (-0.001, 0.003)	0.27	0.001 (-0.0, 0.002)	0.18

Note. Estimated regression coefficients (β), 95% confidence intervals (CI), and *p*-values from the logistic mixed effects models examining whether group (e.g., healthy weight, weight stable [HWWS], weight gain [WG] predicted RNI at year 2 (ages 11/12-years-old). Models were controlled for the caregiver's highest education (effects coded) and framewise displacement. A random intercept was modeled for scanner serial number (e.g., MRI scanner ID). Given that group was time invariant, models were conducted separately for each time point. Significant effects are bolded, where * indicates that the association survived the Benjamini-Hochberg multiple comparison testing correction. BMI = body mass index. ROI=Region-of-interest; H=Hemisphere; L=Left; R=Right.

© 2025 Adise S et al. *JAMA Network Open*.

eTable 19. Results From the Logistic Regression for Model 2 in Females at Baseline

Group (HW-WS vs. HW-NS) ~ Age + RNI + Puberty + Education + Motion + (1|MRI scanner ID)

		**Age**		**RNI**		**Puberty**	
**ROI**	**H**	**β (95% CI)**	***p***	**β (95% CI)**	***p***	**β (95% CI)**	***p***
Accumbens	L	0.425 (0.142, 0.707)	**0.003**	0.171 (0.007, 0.334)	**0.04**	0.38 (0.31, 0.46)	**<0.001**
	R	0.404 (0.12, 0.688)	**0.005**	0.209 (0.046, 0.372)	**0.01**	0.39 (0.31, 0.46)	**<0.001**
Amygdala	L	0.44 (0.159, 0.721)	**0.002**	0.022 (-0.131, 0.176)	0.78	0.39 (0.31, 0.46)	**<0.001**
	R	0.427 (0.144, 0.709)	**0.003**	0.101 (-0.065, 0.267)	0.23	0.39 (0.31, 0.46)	**<0.001**
Caudate	L	0.419 (0.139, 0.7)	**0.003**	0.18 (0.028, 0.331)	**0.02**	0.39 (0.32, 0.47)	**<0.001**
	R	0.41 (0.126, 0.693)	**0.005**	0.163 (0.01, 0.315)	**0.04**	0.38 (0.31, 0.46)	**<0.001**
Hippocampus	L	0.421 (0.139, 0.702)	**0.003**	0.14 (-0.013, 0.293)	0.07	0.38 (0.31, 0.46)	**<0.001**
	R	0.43 (0.148, 0.712)	**0.003**	0.091 (-0.07, 0.252)	0.27	0.38 (0.31, 0.46)	**<0.001**
Pallidum	L	0.426 (0.14, 0.711)	**0.003**	0.12 (-0.057, 0.298)	0.18	0.38 (0.31, 0.46)	**<0.001**
	R	0.4 (0.113, 0.687)	**0.006**	0.141 (-0.043, 0.326)	0.13	0.39 (0.32, 0.47)	**<0.001**
Putamen	L	0.413 (0.126, 0.701)	**0.005**	0.157 (-0.016, 0.33)	0.07	0.39 (0.31, 0.46)	**<0.001**
	R	0.376 (0.088, 0.665)	**0.01**	0.178 (0.009, 0.348)	**0.04**	0.39 (0.31, 0.46)	**<0.001**
Thalamus	L	0.475 (0.193, 0.757)	**0.001**	-0.074 (-0.226, 0.077)	0.34	0.38 (0.31, 0.46)	**<0.001**
	R	0.46 (0.178, 0.741)	**0.001**	-0.007 (-0.163, 0.15)	0.93	0.39 (0.31, 0.47)	**<0.001**
Ventral DC	L	0.455 (0.175, 0.736)	**0.001**	-0.011 (-0.171, 0.148)	0.89	0.39 (0.32, 0.47)	**<0.001**
	R	0.458 (0.177, 0.738)	**0.001**	-0.018 (-0.177, 0.142)	0.83	0.39 (0.31, 0.47)	**<0.001**

Note. Estimated regression coefficients (β), 95% confidence intervals (CI), and *p*-values from the logistic mixed effects models examining the association whether RNI at baseline (ages 9/10-years-old) predicted group membership (e.g., healthy weight, weight stable [HW-WS], [initially] healthy weight, not-stable [HW-NS]). Models were controlled for the caregiver's highest education (effects coded) and framewise displacement. A random intercept was modeled for scanner serial number (e.g., MRI scanner ID). Given that group was time invariant, models were conducted separately for each time point. Significant effects are bolded, where * indicates that the association survived the Benjamini-Hochberg multiple comparison testing correction. BMI = body mass index. ROI=Region-of-interest; H=Hemisphere; L=Left; R=Right.

© 2025 Adise S et al. *JAMA Network Open*.

eTable 20. Results From the Logistic Regression for Model 2 in Females at Year 2

Group (HW-WS vs. HW-NS) ~ RNI + Age + Puberty + Education + Motion + (1|MRI scanner ID)

		Age		RNI		Puberty	
**ROI**	**H**	**β (95% CI)**	***p***	**β (95% CI)**	***p***	**β (95% CI)**	***p***
Accumbens	L	0.301 (0.015, 0.587)	**0.04**	0.138 (-0.022, 0.298)	0.09	0.573 (0.379, 0.768)	**<0.001**
	R	0.317 (0.031, 0.603)	**0.03**	0.148 (-0.01, 0.306)	0.07	0.563 (0.367, 0.759)	**<0.001**
Amygdala	L	0.339 (0.053, 0.625)	**0.02**	-0.005 (-0.162, 0.151)	0.95	0.574 (0.381, 0.767)	**<0.001**
	R	0.339 (0.05, 0.627)	**0.02**	0.021 (-0.143, 0.184)	0.81	0.588 (0.392, 0.785)	**<0.001**
Caudate	L	0.313 (0.029, 0.597)	**0.03**	0.132 (-0.017, 0.28)	0.08	0.57 (0.377, 0.763)	**<0.001**
	R	0.304 (0.019, 0.59)	**0.04**	0.159 (0.002, 0.316)	**0.05**	0.564 (0.37, 0.757)	**<0.001**
Hippocampus	L	0.279 (-0.009, 0.566)	**0.06**	0.219 (0.053, 0.384)	**0.009**	0.587 (0.392, 0.782)	**<0.001**
	R	0.297 (0.009, 0.584)	**0.04**	0.137 (-0.025, 0.3)	0.10	0.578 (0.383, 0.773)	**<0.001**
Pallidum	L	0.296 (0.004, 0.588)	**0.05**	0.071 (-0.106, 0.247)	0.43	0.589 (0.393, 0.785)	**<0.001**
	R	0.308 (0.02, 0.597)	**0.04**	0.048 (-0.124, 0.221)	0.58	0.581 (0.386, 0.777)	**<0.001**
Putamen	L	0.285 (-0.005, 0.576)	**0.05**	0.156 (-0.011, 0.323)	0.07	0.583 (0.389, 0.777)	**<0.001**
	R	0.285 (-0.004, 0.574)	**0.05**	0.11 (-0.059, 0.279)	0.20	0.595 (0.399, 0.791)	**<0.001**
Thalamus	L	0.335 (0.048, 0.622)	**0.02**	-0.021 (-0.185, 0.142)	0.80	0.573 (0.379, 0.767)	**<0.001**
	R	0.339 (0.053, 0.626)	**0.02**	0.013 (-0.163, 0.188)	0.89	0.58 (0.385, 0.775)	**<0.001**
Ventral DC	L	0.323 (0.033, 0.612)	**0.03**	-0.032 (-0.217, 0.153)	0.74	0.598 (0.401, 0.795)	**<0.001**
	R	0.311 (0.025, 0.598)	**0.03**	-0.007 (-0.182, 0.168)	0.94	0.611 (0.415, 0.807)	**<0.001**

Note. Estimated regression coefficients (β), 95% confidence intervals (CI), and *p*-values from the logistic mixed effects models examining whether RNI at year 2 (ages 11/12-years-old) predicted group membership (e.g., healthy weight, weight stable [HW-WS], [initially] healthy weight, not-stable [HW-NS]). Models were controlled for the caregiver's highest education (effects coded) and framewise displacement. A random intercept was modeled for scanner serial number (e.g., MRI scanner ID). Given that group was time invariant, models were conducted separately for each time point. Significant effects are bolded, where * indicates that the association survived the Benjamini-Hochberg multiple comparison testing correction. BMI = body mass index. ROI=Region-of-interest; H=Hemisphere; L=Left; R=Right.

© 2025 Adise S et al. *JAMA Network Open*.

eTable 21. Results for Model 1 for All Youths (Males and Females) Who Were Classified as Healthy Weight, Weight Stable (HW-WS)

RNI ~ BMI*Age*Sex + Puberty + Education + Motion + (1|MRI scanner ID) + (1|subject ID)

		Age		BMI		BMI*Age		BMI*Age*Sex	
**ROI**	**H**	**β (95% CI)**	***p***	**β (95% CI)**	***p***	**β (95% CI)**	***p***	**β (95% CI)**	***p***
Accumbens	L	0.006 (0.005, 0.006)	**<0.001**	0.001 (-0.0, 0.002)	0.06	-0.0 (-0.001, 0.001)	0.68	0.001 (-0.001, 0.003)	0.20
	R	0.006 (0.005, 0.007)	**<0.001**	0.001 (0.0, 0.003)	**0.04**	0.0 (-0.001, 0.001)	0.66	0.001 (-0.001, 0.003)	0.19
Amygdala	L	0.005 (0.004, 0.006)	**<0.001**	0.0 (-0.001, 0.001)	0.64	0.0 (-0.001, 0.001)	0.66	0.001 (-0.0, 0.002)	0.13
	R	0.005 (0.005, 0.006)	**<0.001**	0.001 (-0.0, 0.002)	0.19	0.0 (-0.0, 0.001)	0.52	0.002 (0.0, 0.003)	**0.02**
Caudate	L	0.004 (0.003, 0.004)	**<0.001**	0.001 (-0.0, 0.002)	0.12	-0.001 (-0.001, 0.0)	0.06	0.0 (-0.001, 0.001)	0.99
	R	0.004 (0.003, 0.004)	**<0.001**	0.001 (-0.0, 0.002)	0.06	-0.0 (-0.001, 0.0)	0.26	0.001 (-0.0, 0.003)	0.07
Hippocampus	L	0.004 (0.003, 0.004)	**<0.001**	0.001 (-0.0, 0.002)	0.21	-0.0 (-0.001, 0.001)	0.95	0.001 (-0.0, 0.002)	0.10
	R	0.004 (0.004, 0.005)	**<0.001**	0.001 (0.0, 0.002)	**0.003***	-0.0 (-0.001, 0.0)	0.33	0.001 (0.0, 0.003)	**0.004**
Pallidum	L	0.015 (0.014, 0.016)	**<0.001**	0.002 (0.0, 0.004)	**0.03**	0.0 (-0.001, 0.001)	0.86	-0.0 (-0.002, 0.002)	0.89
	R	0.015 (0.014, 0.016)	**<0.001**	0.001 (-0.001, 0.003)	0.16	-0.0 (-0.001, 0.001)	0.86	-0.0 (-0.002, 0.002)	0.88
Putamen	L	0.006 (0.005, 0.006)	**<0.001**	0.001 (0.0, 0.002)	**0.02**	0.0 (-0.0, 0.001)	0.76	0.0 (-0.001, 0.001)	0.52
	R	0.006 (0.005, 0.007)	**<0.001**	0.001 (0.0, 0.002)	**0.01**	0.0 (-0.0, 0.001)	0.64	0.0 (-0.001, 0.001)	0.48
Thalamus	L	0.006 (0.005, 0.006)	**<0.001**	0.001 (-0.0, 0.002)	0.23	-0.0 (-0.001, 0.0)	0.23	0.001 (-0.0, 0.002)	0.13
	R	0.006 (0.006, 0.007)	**<0.001**	0.001 (-0.0, 0.002)	0.09	0.0 (-0.001, 0.001)	0.93	0.002 (0.0, 0.003)	**0.02**
Ventral DC	L	0.005 (0.005, 0.006)	**<0.001**	0.001 (0.0, 0.002)	**0.02**	0.0 (-0.001, 0.001)	0.57	0.001 (-0.001, 0.002)	0.29
	R	0.005 (0.005, 0.006)	**<0.001**	0.002 (0.001, 0.003)	**0.004***	-0.0 (-0.001, 0.001)	0.63	0.002 (0.0, 0.003)	**0.03**

Note. Estimated regression coefficients (β), 95% confidence intervals (CI), and *p*-values from the linear mixed-effects models examining whether there were differences in sex on the rate of change of BMI on RNI. Models were controlled for the caregiver's highest education (effects coded) and framewise displacement. Random effects were modeled from subject ID and scanner serial number (e.g., MRI scanner ID). Significant effects are bolded, where * indicates that the association survived the Benjamini-Hochberg multiple comparison testing correction. BMI = body mass index. ROI=Region-of-interest; H=Hemisphere; L=Left; R=Right.

© 2025 Adise S et al. *JAMA Network Open*.

eTable 22. Results for Model 2 for All Youths (Males and Females) Classified as Healthy Weight, Weight Stable (HW-WS)

BMI ~ RNI*Age*Sex + Puberty + Education + Motion + (1|MRI scanner ID) + (1|subject ID)

		Age		RNI		RNI*Age		RNI*Age*Sex	
**ROI**	**H**	**β (95% CI)**	***p***	**β (95% CI)**	***p***	**β (95% CI)**	***p***	**β (95% CI)**	***p***
Accumbens	L	0.38 (0.35, 0.42)	**<0.001**	0.02 (-0.02, 0.07)	0.31	0.0 (-0.02, 0.03)	0.68	0.03 (-0.02, 0.08)	0.21
	R	0.38 (0.34, 0.42)	**<0.001**	0.03 (-0.01, 0.08)	0.14	0.0 (-0.02, 0.03)	0.80	0.03 (-0.02, 0.08)	0.24
Amygdala	L	0.38 (0.34, 0.41)	**<0.001**	0.04 (-0.0, 0.08)	0.07	0.01 (-0.01, 0.04)	0.34	0.03 (-0.02, 0.08)	0.19
	R	0.38 (0.34, 0.41)	**<0.001**	0.04 (-0.01, 0.08)	0.10	0.02 (-0.0, 0.05)	0.06	0.05 (0.0, 0.1)	**0.04**
Caudate	L	0.38 (0.35, 0.42)	**<0.001**	0.02 (-0.02, 0.06)	0.40	-0.02 (-0.05, 0.0)	0.09	-0.01 (-0.06, 0.04)	0.76
	R	0.39 (0.35, 0.42)	**<0.001**	0.01 (-0.03, 0.05)	0.53	-0.02 (-0.04, 0.01)	0.21	0.02 (-0.03, 0.07)	0.38
Hippocampus	L	0.38 (0.35, 0.42)	**<0.001**	0.03 (-0.01, 0.08)	0.17	0.01 (-0.02, 0.03)	0.50	0.01 (-0.04, 0.06)	0.75
	R	0.37 (0.33, 0.4)	**<0.001**	0.07 (0.02, 0.12)	**0.004**	0.01 (-0.02, 0.03)	0.53	0.02 (-0.02, 0.07)	0.33
Pallidum	L	0.37 (0.33, 0.41)	**<0.001**	0.04 (-0.01, 0.09)	0.15	-0.0 (-0.03, 0.02)	0.88	0.02 (-0.03, 0.07)	0.45
	R	0.38 (0.34, 0.42)	**<0.001**	0.02 (-0.03, 0.08)	0.42	-0.02 (-0.04, 0.01)	0.19	0.0 (-0.04, 0.05)	0.89
Putamen	L	0.37 (0.33, 0.41)	**<0.001**	0.04 (-0.01, 0.09)	0.13	0.0 (-0.02, 0.02)	1.00	0.03 (-0.02, 0.08)	0.28
	R	0.36 (0.33, 0.4)	**<0.001**	0.05 (0.0, 0.1)	**0.04**	-0.0 (-0.03, 0.02)	0.96	0.04 (-0.01, 0.09)	0.11
Thalamus	L	0.38 (0.35, 0.42)	**<0.001**	0.02 (-0.03, 0.07)	0.38	-0.01 (-0.04, 0.01)	0.37	0.01 (-0.04, 0.05)	0.78
	R	0.38 (0.34, 0.41)	**<0.001**	0.04 (-0.01, 0.08)	0.16	-0.01 (-0.03, 0.02)	0.55	0.01 (-0.04, 0.05)	0.82
Ventral DC	L	0.37 (0.34, 0.41)	**<0.001**	0.05 (0.01, 0.1)	**0.02**	0.0 (-0.02, 0.02)	0.98	-0.0 (-0.05, 0.05)	0.99
	R	0.37 (0.34, 0.41)	**<0.001**	0.05 (0.01, 0.1)	**0.03**	0.01 (-0.02, 0.03)	0.64	0.0 (-0.04, 0.05)	0.85

Note. Estimated regression coefficients (β), 95% confidence intervals (CI), and *p*-values from the linear mixed-effects models examining whether differences in sex are related to the rate of change in RNI on leading to changes in BMI. Models were controlled for the caregiver's highest education (effects coded) and framewise displacement. Random effects were modeled from subject ID and scanner serial number (e.g., MRI scanner ID). Significant effects are bolded, where * indicates that the association survived the Benjamini-Hochberg multiple comparison testing correction. Puberty showed significant positive associations with BMI, but had less of an association than age (β=0.14, $p<0.01$). BMI = body mass index. ROI=Region-of-interest; H=Hemisphere; L=Left; R=Right.

© 2025 Adise S et al. *JAMA Network Open*.

eTable 23. Results for Model 1 for All Youths (Males and Females) With Various Amounts of Weight Gain (ie, Relatively Weight Stable to Relatively Unhealthy Weight Gain)

RNI ~ BMI*Age*Sex + Puberty + Education + Motion + (1|MRI scanner ID) + (1|subject ID)

		Age		BMI		BMI*Age		BMI*Age*Sex	
**ROI**	**H**	**β (95% CI)**	***p***	**β (95% CI)**	***p***	**β (95% CI)**	***p***	**β (95% CI)**	***p***
Accumbens	L	0.005 (0.005, 0.006)	**<0.001**	0.002 (0.001, 0.003)	**<0.001***	-0.0 (-0.001, 0.0)	0.38	-0.0 (-0.001, 0.001)	0.98
	R	0.006 (0.005, 0.006)	**<0.001**	0.002 (0.001, 0.002)	**<0.001***	-0.0 (-0.001, 0.0)	0.35	-0.0 (-0.001, 0.001)	0.51
Amygdala	L	0.005 (0.004, 0.005)	**<0.001**	0.0 (-0.0, 0.001)	0.48	-0.0 (-0.0, 0.0)	0.56	0.001 (-0.0, 0.001)	0.15
	R	0.005 (0.005, 0.006)	**<0.001**	0.001 (-0.0, 0.001)	0.06	-0.0 (-0.0, 0.0)	0.62	0.0 (-0.0, 0.001)	0.33
Caudate	L	0.004 (0.003, 0.004)	**<0.001**	0.001 (0.001, 0.002)	**<0.001***	-0.0 (-0.001, -0.0)	0.04	-0.0 (-0.001, 0.0)	0.46
	R	0.004 (0.003, 0.004)	**<0.001**	0.001 (0.001, 0.002)	**<0.001***	-0.001 (-0.001, -0.0)	**0.003***	-0.0 (-0.001, 0.001)	0.83
Hippocampus	L	0.004 (0.003, 0.004)	**<0.001**	0.001 (0.0, 0.001)	**0.001***	-0.0 (-0.0, 0.0)	0.15	0.0 (-0.0, 0.001)	0.54
	R	0.004 (0.004, 0.005)	**<0.001**	0.001 (0.0, 0.001)	**<0.001***	-0.0 (-0.0, 0.0)	0.17	0.001 (-0.0, 0.001)	0.06
Pallidum	L	0.014 (0.013, 0.015)	**<0.001**	0.002 (0.001, 0.003)	**<0.001***	-0.0 (-0.001, 0.0)	0.19	0.0 (-0.001, 0.001)	0.95
	R	0.014 (0.013, 0.015)	**<0.001**	0.002 (0.001, 0.003)	**<0.001***	-0.001 (-0.001, 0.0)	**0.05**	-0.001 (-0.002, 0.0)	0.29
Putamen	L	0.006 (0.005, 0.006)	**<0.001**	0.001 (0.001, 0.001)	**<0.001***	-0.0 (-0.001, -0.0)	**0.005***	0.0 (-0.0, 0.001)	0.71
	R	0.006 (0.005, 0.006)	**<0.001**	0.001 (0.001, 0.001)	**<0.001***	-0.0 (-0.001, -0.0)	**0.03**	-0.0 (-0.001, 0.001)	0.93
Thalamus	L	0.006 (0.005, 0.006)	**<0.001**	0.001 (0.001, 0.002)	**<0.001***	-0.0 (-0.001, -0.0)	**0.007***	0.0 (-0.0, 0.001)	0.18
	R	0.006 (0.006, 0.007)	**<0.001**	0.001 (0.001, 0.002)	**<0.001***	-0.0 (-0.001, -0.0)	**0.03**	0.001 (0.0, 0.002)	0.04
Ventral DC	L	0.005 (0.005, 0.006)	**<0.001**	0.001 (0.0, 0.001)	**0.001**	-0.0 (-0.0, 0.0)	0.69	0.0 (-0.0, 0.001)	0.43
	R	0.005 (0.005, 0.006)	**<0.001**	0.001 (0.0, 0.002)	**<0.001***	-0.0 (-0.001, 0.0)	0.16	0.0 (-0.0, 0.001)	0.25

Note. Estimated regression coefficients (β), 95% confidence intervals (CI), and *p*-values from the linear mixed-effects models examining whether differences in sex led to a change in BMI on RNI. Models were controlled for the caregiver's highest education (effects coded) and framewise displacement. Random effects were modeled from subject ID and scanner serial number (e.g., MRI scanner ID). Significant effects are bolded, where * indicates that the association survived the Benjamini-Hochberg multiple comparison testing correction. BMI = body mass index. ROI=Region-of-interest; H=Hemisphere; L=Left; R=Right.

© 2025 Adise S et al. *JAMA Network Open*.

eTable 24. Results for Model 2 for All Youths (Males and Females) With Various Amounts of Weight Gain (ie, Relatively Weight Stable to Relatively Unhealthy Weight Gain)

BMI ~ RNI*Age*Sex + Puberty + Education + Motion + (1|MRI scanner ID) + (1|subject ID)

		Age		RNI		RNI*Age		RNI*Age*Sex	
**ROI**	**H**	**β (95% CI)**	***p***	**β (95% CI)**	***p***	**β (95% CI)**	***p***	**β (95% CI)**	***p***
Accumbens	L	0.74 (0.69, 0.78)	**<0.001**	0.16 (0.11, 0.21)	**<0.001***	0.07 (0.04, 0.11)	**<0.001***	0.12 (0.05, 0.19)	**0.001***
	R	0.74 (0.69, 0.79)	**<0.001**	0.13 (0.08, 0.18)	**<0.001***	0.07 (0.04, 0.11)	**<0.001***	0.11 (0.04, 0.18)	**0.002***
Amygdala	L	0.78 (0.74, 0.83)	**<0.001**	0.02 (-0.03, 0.07)	0.48	0.03 (-0.01, 0.06)	0.12	0.09 (0.02, 0.16)	**0.01**
	R	0.77 (0.72, 0.82)	**<0.001**	0.04 (-0.01, 0.09)	0.12	0.05 (0.01, 0.08)	**0.01***	0.07 (0.0, 0.14)	0.04
Caudate	L	0.76 (0.72, 0.81)	**<0.001**	0.09 (0.04, 0.14)	**<0.001***	0.03 (-0.01, 0.06)	0.15	0.05 (-0.02, 0.12)	0.18
	R	0.76 (0.71, 0.8)	**<0.001**	0.11 (0.06, 0.16)	**<0.001***	0.02 (-0.01, 0.06)	0.19	0.12 (0.05, 0.2)	**0.001***
Hippocampus	L	0.76 (0.72, 0.81)	**<0.001**	0.08 (0.03, 0.14)	**0.001***	0.03 (-0.01, 0.07)	0.10	0.11 (0.04, 0.18)	**0.003***
	R	0.75 (0.7, 0.8)	**<0.001**	0.09 (0.04, 0.14)	**0.001***	0.02 (-0.02, 0.05)	0.30	0.1 (0.03, 0.17)	**0.004***
Pallidum	L	0.73 (0.68, 0.78)	**<0.001**	0.12 (0.06, 0.18)	**<0.001***	0.05 (0.01, 0.08)	**0.009***	0.12 (0.05, 0.19)	**0.001***
	R	0.74 (0.69, 0.8)	**<0.001**	0.08 (0.02, 0.14)	**0.006***	0.02 (-0.01, 0.06)	0.18	0.07 (0.0, 0.14)	**0.05**
Putamen	L	0.73 (0.68, 0.78)	**<0.001**	0.11 (0.05, 0.16)	**<0.001***	0.06 (0.02, 0.1)	**0.001***	0.15 (0.08, 0.22)	**0.001***
	R	0.73 (0.68, 0.78)	**<0.001**	0.1 (0.05, 0.15)	**<0.001***	0.06 (0.02, 0.09)	**0.002***	0.13 (0.06, 0.2)	**0.001***
Thalamus	L	0.77 (0.72, 0.82)	**<0.001**	0.07 (0.01, 0.12)	**0.02***	-0.01 (-0.04, 0.03)	0.63	0.06 (-0.01, 0.14)	0.08
	R	0.76 (0.71, 0.81)	**<0.001**	0.09 (0.03, 0.15)	**0.002***	0.01 (-0.03, 0.05)	0.57	0.12 (0.04, 0.19)	**0.002***
Ventral DC	L	0.76 (0.72, 0.81)	**<0.001**	0.07 (0.02, 0.13)	**0.01***	0.02 (-0.01, 0.06)	0.21	0.04 (-0.03, 0.12)	0.22
	R	0.77 (0.72, 0.82)	**<0.001**	0.07 (0.01, 0.12)	**0.02***	0.01 (-0.02, 0.05)	0.44	0.05 (-0.03, 0.12)	0.21

Note. Estimated regression coefficients (β), 95% confidence intervals (CI), and *p*-values from the linear mixed-effects models examining whether differences in sex led to changes in RNI on BMI. Models were controlled for the caregiver's highest education (effects coded) and framewise displacement. Random effects were modeled from subject ID and scanner serial number (e.g., MRI scanner ID). Significant effects are bolded, where * indicates that the association survived the Benjamini-Hochberg multiple comparison testing correction. Puberty showed significant positive associations with BMI but had less of an association than age (β=0.31, $p<0.01$). BMI = body mass index. ROI=Region-of-interest; H=Hemisphere; L=Left; R=Right.

© 2025 Adise S et al. *JAMA Network Open*.
