# Supplementary material for: Distinct Patterns of Weight Gain, Age, and Subcortical Microstructure in Early Adolescence
Source: JAMA Netw Open. 2025 Jul 23;8(7):e2522211. doi: 10.1001/jamanetworkopen.2025.22211 (PMC12287857; doi:10.1001/jamanetworkopen.2025.22211)
Supplement: Supplement 2. — Data Sharing Statement [file jamanetwopen-e2522211-s002.pdf]

Data Sharing Statement

Adise. Distinct Patterns of Weight Gain, Age, and Subcortical Microstructure in Early Adolescence. *JAMA Netw Open*. Published July 23, 2025.
doi:10.1001/jamanetworkopen.2025.22211

Data
Data available: No

Additional Information
Explanation for why data not available: it is already but you need your own data use agreement per NDA
